# Methods to Study the Molecular Mechanism and Drive the Design of Protein Degraders

**DOI:** 10.1021/acs.chemrev.5c00800

**Published:** 2026-05-14

**Authors:** Charlotte Crowe, Alessio Ciulli

**Affiliations:** Centre for Targeted Protein Degradation, School of Life Sciences, 3042 University of Dundee, 1 James Lindsay Place, Dundee DD1 5JJ, U.K.

## Abstract

Small-molecule degraders eliminate disease-driving proteins by hijacking the ubiquitin-proteasome system. To achieve cellular activity, protein degraders must perform a series of consecutive steps involving cell permeability, binary target engagement, and formation of a ternary complex with the target protein and a ubiquitin E3 ligase, followed by protein ubiquitination, culminating with protein degradation. Monitoring each mechanistic step of a degraders’ mode of action is important to confirm its *bona fide* cellular activity and guide rational design and optimization. In this review, we offer an overview of how degraders work and outline the key parameters and associated methods to study each step of the mechanism. We compare and contrast biophysical and cellular *in vitro* assays and provide a concise framework for prioritizing and mapping them to decision stages. We also discuss the main factors affecting degrader’s cellular performance and principles that have emerged to guide drug design.

## Graphical abstract

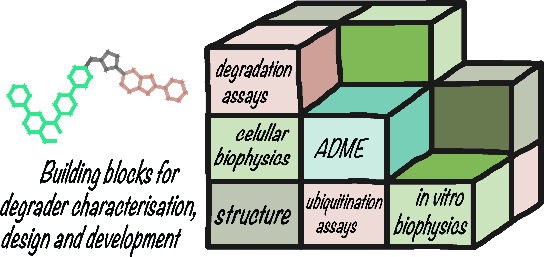

## 1 Introduction

Targeted protein degradation (TPD) has emerged as a powerful therapeutic strategy for degrading proteins implicated in disease. In TPD, small molecules are used to harness the activity of the ubiquitin-proteasome system (UPS), for example, inducing ubiquitination and subsequent degradation of a *neo*-substrate protein. This is most commonly achieved by small molecules that bind to an E3 ligase and then recruit the target protein to the E3. These molecules, also called protein degraders, are typically either bivalent as is the case for proteolysis targeting chimeras (PROTACs) which can engage to the E3 and target protein separately first and then simultaneously as a ternary complex (Figure 1 **A**),^1^ or which preferentially bind to either E3 or target, as is the case for the so-called monovalent “molecular glues” which then induce recruitment of the other protein (Figure 1 **B**).^2^ More recently, degraders with different structures and binding features have been explored which do not operate through these two canonical categories (Figure 1 **C**).^3,4^

**Figure 1:**
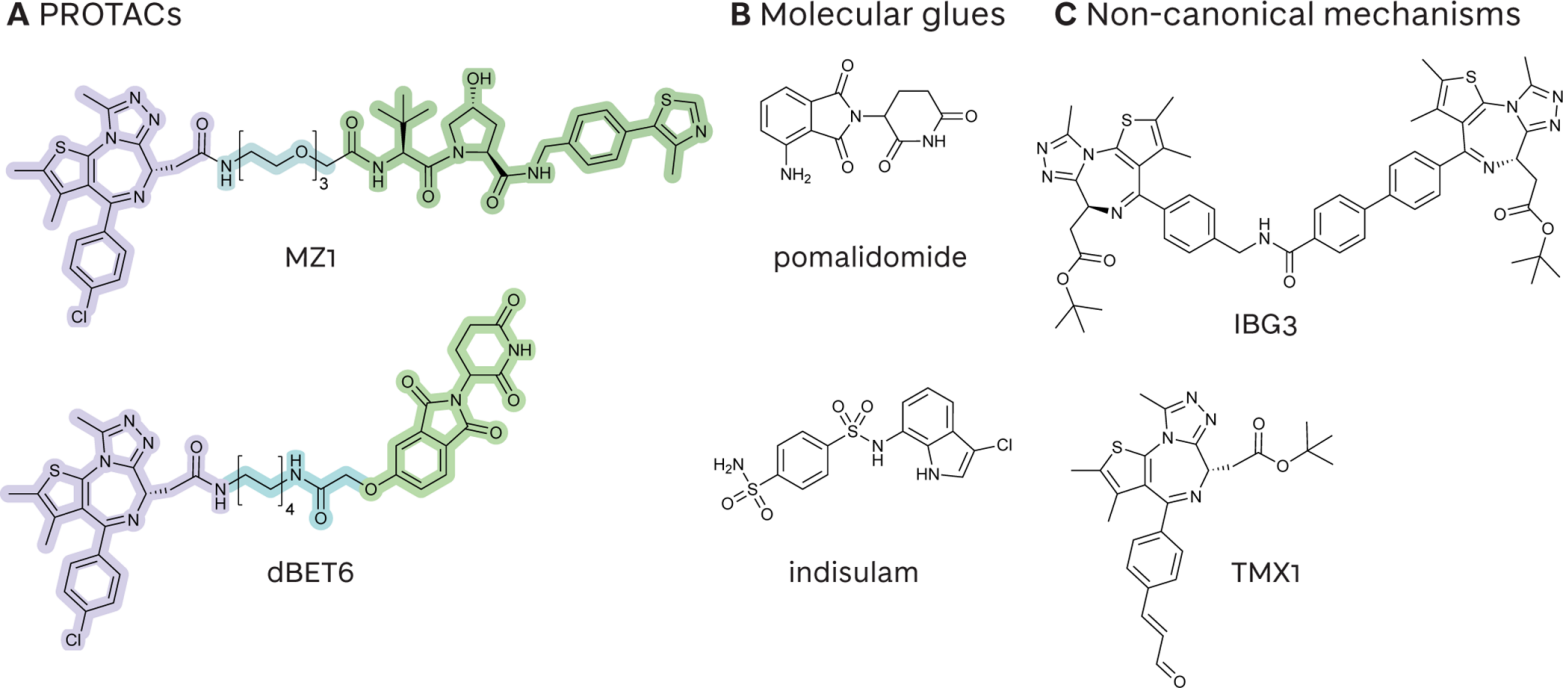
Examples of widely studied and mechanistically diverse small molecule degraders. **A** PROTACs: MZ1 and dBET6, with highlighted target protein binding moiety (purple), linker region (blue), and E3 ligase binding moiety (green);^5,6^ **B** Monovalent molecular glues: pomalidomide and indisulam; **C** Examples of degraders which recruit the target protein and E3 ligase in a ternary complex via noncanonical mechanisms, blurring the conventional boundaries between PROTACs and molecular glues: intramolecular bivalent glues and template-assisted covalent modifiers.^3,4^

Until recently, the majority of small molecule drugs aimed to occupy a protein binding site and block its functionality by acting as an inhibitor, receptor antagonist, or protein–protein interaction (PPi) disruptor. High sustained cellular levels of the drug agent are required to achieve and maintain the desired pharmacological response. In contrast, small molecule degraders can offer catalytic efficacy requiring only substoichiometric cellular levels of drug relative to its protein target. In addition, degraders do not need to bind a functional site on the protein as they can bind anywhere on the target protein to exert their ubiquitination activity, presenting the opportunity to tackle so-called “un-druggable” targets. Another immediate advantage of TPD strategies is that the downstream result of their action is the actual depletion of the targeted protein, which phenocopies and mechanistically mimics much more closely the effect of genetic knockouts or knock-down. Degrading a whole protein is also very different from blockade of a single binding site and can impact also on scaffolding functions.

Owing to these attractive advantages over occupancy-based modalities, TPD has become a rapidly expanding research field. Since their first proposition in 2001,^1^ and their breakthrough development and validation in cells and *in vivo* in 2015,^5,7–9^ PROTAC degraders have been developed to work via recruiting a repertoire of E3 ligases,^3,10–14^ prevalently to-date the Cullin 2 RING von Hippel-Lindau (CRL2^VHL^),^15–17^ and the Cullin 4 RING cereblon (CRL4^CRBN^),^18–20^ and targeting a large variety of disease-causing proteins, such as (and not limited to) protein kinases,^21^ nuclear receptors,^22^ epigenetic modulators,^23^ cell-surface receptors,^24^ transcription factors,^25^ antiapoptotic proteins,^26^ proteins responsible for neurodegenerative diseases,^27^ and virus-related proteins.^28^

The advancement of two PROTAC molecules, ARV-110 and ARV-471 (see Note added in Proof), into clinical trials against prostate and breast cancer in 2019–2021 constituted a milestone in the field.^29^ As of 2025, there are >50 degrader drug candidates that are currently in clinical trials for various diseases, collectively between PROTACs and molecular glues.^30,31^ To guide the design of future degrader drugs in the clinic, the field has developed and applied a wide palette of diverse and robust assays informing on the individual steps of the mechanisms whereby degraders act.

In this review, we provide a comprehensive overview of the key parameters and current technologies for characterizing and evaluating the efficacy of small molecule degraders that are employed for targeted degradation of proteins via the ubiquitin proteasome system, from the inception of the TPD field to the present date. While several of these technologies have been covered in previous reviews,^32,33^ the rapid exponential growth of the TPD field warrants an up-to-date summary and appraisal of methods which have been applied to date. We cover methodology relating to the study of small molecule degraders *in vitro* and in cells, to characterize cellular permeability and intracellular availability, binary binding, ternary complex formation, ubiquitination, and degradation. This review will not involve in-depth discussion of *in vivo* or clinical applications, nor of proteomic or computational approaches, as several of these topics have recently been covered elsewhere.^34–36^

## 2 Mechanism and Parameters for Evaluating Small Molecule Degrader Efficacy

Because small molecule degraders redirect the E3 ligase to ubiquitinate a *neo*-substrate protein, they follow a catalytic cycle. First, the PROTAC must enter the cell, then engage separately with the E3 ligase or the *neo-*substrate protein, before engaging the two proteins simultaneously to form the key ternary complex species which enables ubiquitination and degradation (Figure 2). Following degradation by the 26S proteasome, the degrader molecule can be released from its protein complex and recycled, free to undergo another cycle of catalysis.

**Figure 2:**
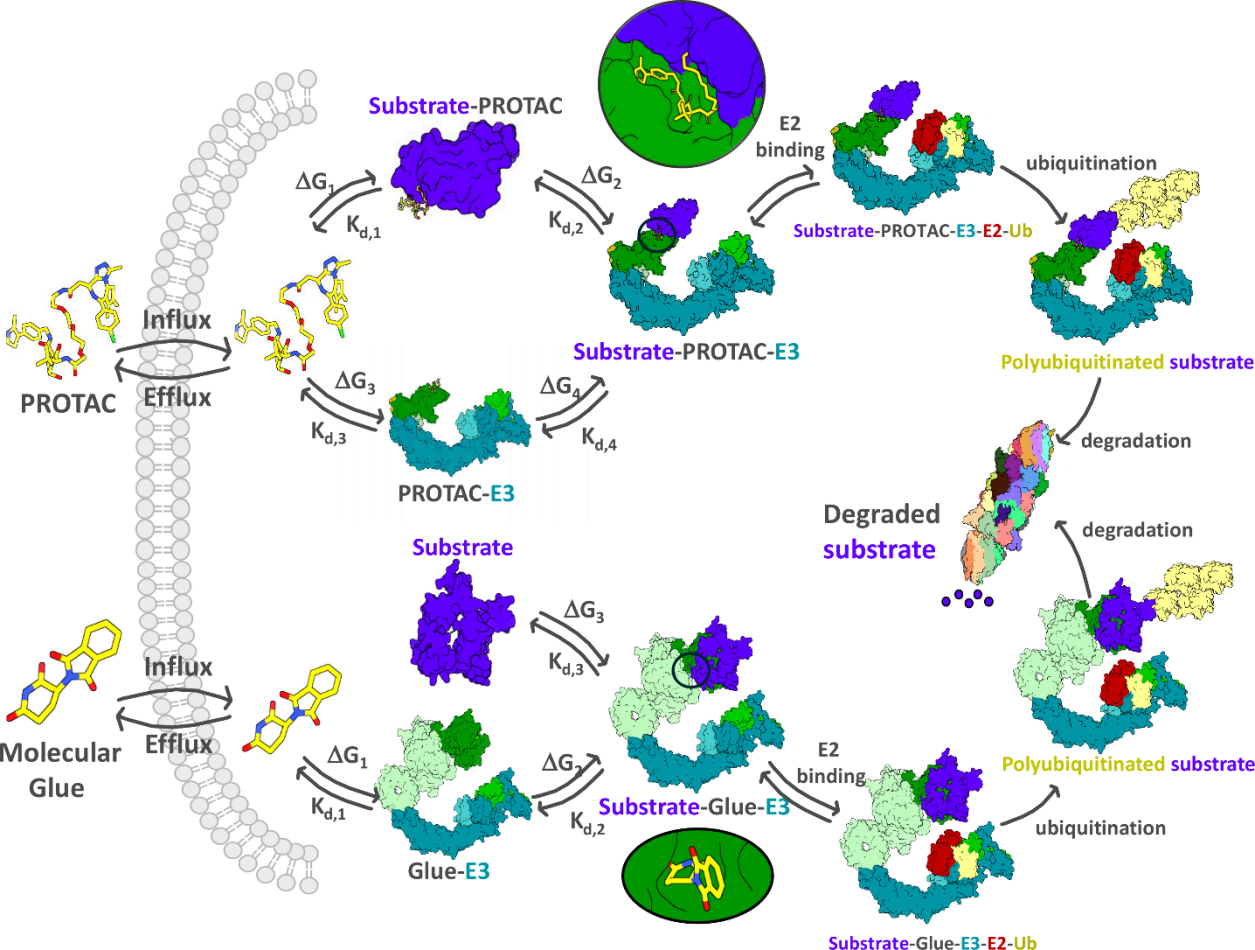
Representation of the key steps for targeted protein degradation mediated by a PROTAC (above pathway) or a molecular glue (below pathway) degrader. The degrader (yellow, colored by heteroatom), substrate (purple), E3 ligase (shades of blue and green), E2 (red), ubiquitin (yellow), and the 26S proteasome (multicolored) are shown.

PROTACs are typically larger in size than molecular glues, with many exceeding 1000 Da, which poses a challenge for optimizing their pharmacokinetic properties while retaining excellent activity in vivo. PROTACs, in particular, as ‘beyond rule of 5’ (bRo5) molecules, can be designed and characterized by a wealth of descriptors relating to the degrader’s size, lipophilicity, ionization, polarity, and chameleonicity, all governing the molecules’ ability to enter the cell and adequately biodistribute when dosed in vivo (Table 1).^37^

**Table 1:** Overview of the Parameters and Values Governing the Chemical Design of Efficacious Degraders for Cellular Permeability

*Parameter*	*Description*	*‘Favorable’ values*	*Effect on degrader performance*
*MW*	Molecular weight	<950 Da (ref.^38^); <1000 (ref.^39^)	Size
*nC*	Number of carbons	^a^	Size
*LogP*	Calculated lipophilicity partition coefficient	1–7 (ref.^38^); <5 (ref.^54^)	Lipophilicity
*LogD*	Lipophilicity distribution coefficient of ionizable compounds	0–6 (ref.^38^)	Lipophilicity
*BRlogP/BRDlogD*	Experimental ‘biologically relevant logP/logD’	2 to 5 (ref.^43^)	Lipophilicity
LogS	Thermodynamic solubility	>200 μM^48^	Solubility
*pK*_*a*_	Acid–base dissociation constant	^a^	Ionizability
*log* k*’80* PLRP-S	Chromatographic retention factor measured on a polystyrene/divinylbenzene polymeric column with 80% aprotic acetonitrile in the mobile phase	^a^	Ionizability
*NAr*	Number of aromatic groups	<5 (ref.^38^)	Lipophilicity, stacking
*CHI*	Chromatographic hydrophobicity index	^a^	Lipophilicity
*ElogP/ElogD*	Estimated logP/logD	^a^	Lipophilicity
*ChromLogD*	Chromatographic logD	4.4–6.8 (ref.^55^); ≤7 (ref.^39^)	Lipophilicity
*HBD*	Number of hydrogen bond donors	≤2 (ref.^38^)	Polarity
*HBA*	Number of hydrogen bond acceptors	15 (ref.^38^)	Polarity
*TPSA*	Total polar surface area	≤200 Å^2^ (ref.^38^)	Polarity
*EPSA*	Experimental polar surface area	≤170 Å^2^ (ref.^39^)	Polarity
*Δlog k*_*W*_^*IAM*^	immobilized artificial membrane (IAM) chromatographic retention factor	^a^	Polarity
*eHBD*	Solvent-exposed H-bond donors	≤2 (ref.^39^)	Polarity
*eHBA*	Solvent-exposed H-bond acceptors	≤16 (ref.^39^)	Polarity
*Chamelogk*	Chameleonicity metric	^a^	Chameleonicity, shape, flexibility
*CharVol*	Characteristic volume	^a^	Chameleonicity, shape, flexibility
*NRotB*	Number of rotatable bonds	≤14 (ref.^38^); ≤13 (ref.^39^)	Chameleonicity, shape, flexibility
*PHI*	Kier’s flexibility index	^a^	Flexibility
*R*_*gyr*_	Radius of gyration	^a^	Chameleonicity, shape, flexibility

a A specific cutoff has not been defined in the literature.

Too large a compound size, described by molecular weight (MW) and number of carbons (nC), can hinder cellular entry.^38,39^ Lipophilicity parameters, classically described by the calculated lipophilicity partition coefficient (LogP) and the lipophilicity distribution coefficient of ionizable compounds (LogD), are considered a major factor for cell permeability. Several descriptors have been developed to quantify this, such as the chromatographic hydrophobicity index (CHI),^40^ ElogP,^41^ ElogD,^42^ and the experimental ‘biologically relevant logP’ (*BR*logP)^43^ and ‘biologically relevant logD’ (*BR*logD).^44^

Polarity constitutes another hurdle for degrader molecules, with too high polarity leading to desolvation penalties that prohibit membrane entry. While the number of hydrogen bond donors (HBD) and hydrogen bond acceptors (HBA) are classically used to characterize compound polarity, more recently experimental descriptors of solvent-exposed H-bond donors (eHBD) and acceptors (eHBA) have been proposed.^39^ Furthermore, minimizing the total polar surface area (TPSA) and the experimental polar surface area (EPSA)^45^ are also critical for enhancing cellular permeability. The Δlog k_W_^IAM^ value, which has been shown to be a key descriptor for bRo5 compounds, relates to the immobilized artificial membrane (IAM) chromatographic retention factor.^46,47^

Another key parameter for degrader efficacy is solubility, which is governed by lipophilicity and polarity.^48^ Not only can this influence cellular permeability, but low degrader solubility can also confound or prevent reliable experimental results.^49^ A key parameter is the thermodynamic solubility (LogS), where ‘high solubility’ degrader molecules are classed as >200 μM.

Given that bRo5 compounds such as PROTACs can be ionizable, ionization is a key property contributing to degrader cellular permeability. Classically, the acid–base dissociation constant (p*K*_*a*_) is used to describe ionization; however, in such cases where the p*K*_*a*_ cannot be determined due to low compound solubility or other experimental challenges, the chromatographic descriptor log *k*’80 PLRP-S at different pH values can estimate ionization properties.^50^

More recently, the shape, chameleonicity, and flexibility of PROTAC degraders have been explored, with further parameters shown to influence membrane permeability such as the chameleonicity metric (Chamelogk),^51^ the characteristic volume (CharVol),^52^ and the number of rotatable bonds (NRotB).^53^

Together, these parameters give indications to predict a PROTAC or molecular glue cellular permeability.

As PROTACs are heterobifunctional molecules with a ‘modular’ structure, they have distinct ligand moieties for the target protein and E3 ligase and a distinct linker region [Figure 1 **A** with highlighted binding moiety (purple), linker region (blue), and E3 ligase binding moiety (green)]. Therefore, they can simultaneously or asynchronously interact with the *neo*-substrate and the E3 ligase (Figure 2). The free energy change of forming the ternary complex ΔG_complex_ can be described by eq (1) (Figure 3). A key parameter which emerges from this equilibrium treatment is cooperativity (α), defined by the ratio of a PROTAC’s binary to ternary dissociation constant K_d_ [eq (2), Figure 3].^56^ When α = 1, the system is noncooperative and the binding affinity for one or the other partner protein remains unaltered irrespective of whether it involves forming the binary complex or the ternary complex. Values of α < 1 describe a negatively cooperative system, where the equilibrium of the system is shifted toward favoring binary over ternary binding. If α > 1, the system is described as positively cooperative because it favors the formation of a ternary complex over binary binding. Positive cooperativity coupled with high-affinity for ternary complex formation typically provides a favorable scenario for driving ternary complex stability, enhanced ubiquitination, and efficient degradation.^16,57–63^

**Figure 3:**
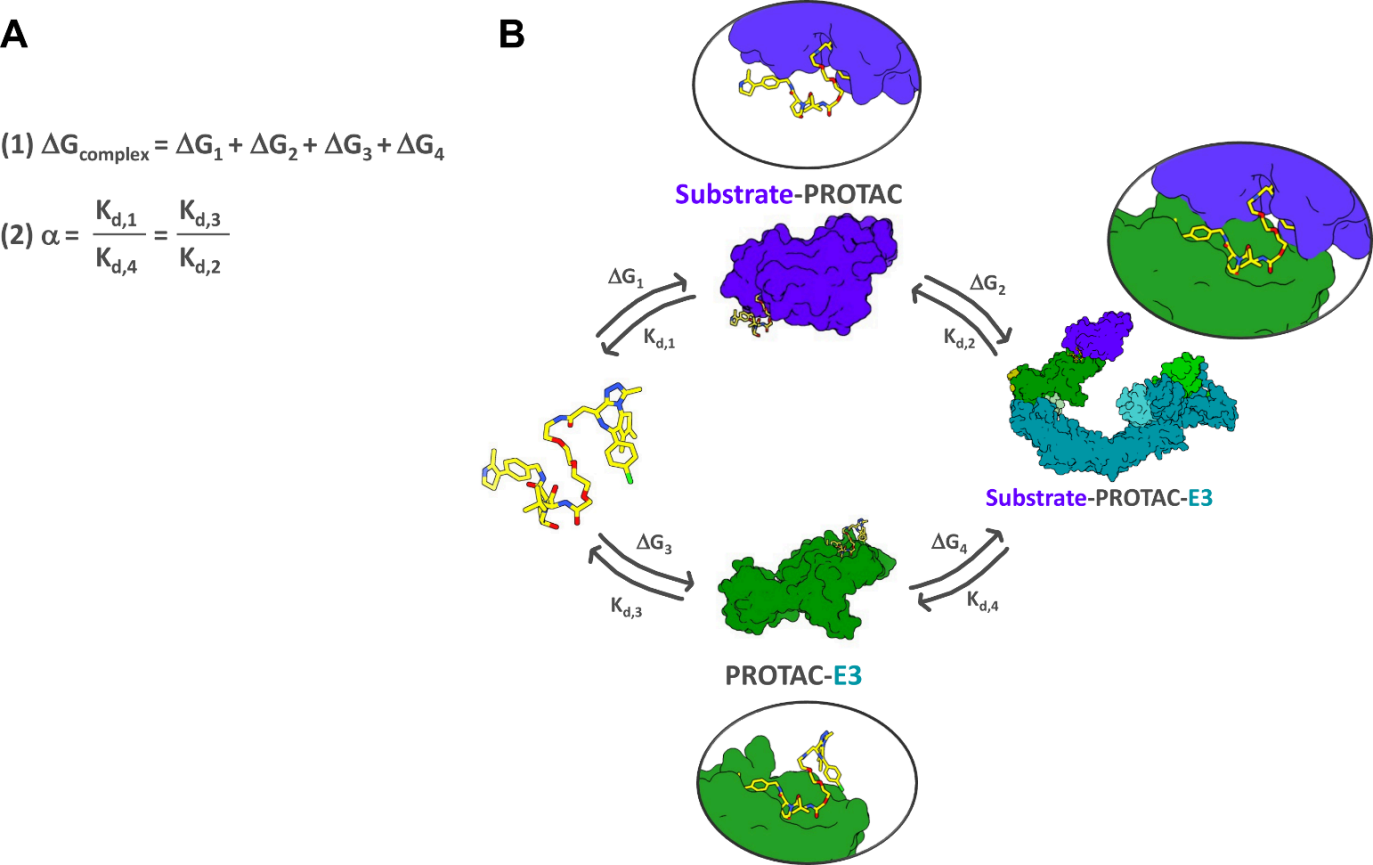
Equilibria of PROTAC-mediated ternary complex formation. **A** (1) Equation describing the free energy change of forming the ternary complex ΔG_complex_ and (2) equation describing the cooperativity value α. **B** The three-body equilibrium model describing PROTAC binary binding and ternary complex formation.

A phenomenon exhibited by PROTACs and indeed any bifunctional molecules is the so-called ‘hook effect’. First observed in Western blotting, where protein ubiquitination and degradation levels were reduced at higher PROTAC concentrations,^9,64^ the ‘hook effect’ describes a scenario where the PROTAC is in too high concentration relative to the target and E3; therefore, ternary complex formation is reduced because it is competed in favor of binary binding with the respective partners. The hook effect occurs readily for negatively cooperative and noncooperative PROTACs, and particularly for PROTACs made of high-affinity ligands, but it is alleviated for positively cooperative PROTACs, and delayed until very high compound concentrations, often unachievable in cells and in vivo.

In addition to cooperativity, the favorability of the substrate-PROTAC-E3 complex is also dependent on its longevity and stability. Thermodynamic studies can be used to determine the ternary complex’s dissociation constant K_d_ (and the corresponding association constant K_a_ = 1/K_d_), including binding enthalpy ΔH and entropy ΔS, as well as the binding stoichiometry coefficient N, while kinetic measurements allow to attain additional information on on-rate *k*_on_, off-rate *k*_off_, and half-life *t*_1/2_ of the complex.

Molecular glue degraders use the same cellular degradation machinery and follow a similar catalytic cycle to PROTACs (Figure 2). They typically have a lower molecular weight and therefore more favorable ‘drug-like’ properties which allow for more facile cell penetration and potentially increased oral bioavailability. A key mechanistic difference is that molecular glues typically interact with only one of the two proteins at binary level: for example, for the archetypical molecular glue degrader thalidomide, it interacts exclusively with the E3 ligase CRBN, and once that binary complex is formed, it can induce or stabilize recruitment of a *neo*-substrate.^65^ For this reason, the recruitment of the second protein in the presence of the glue must be highly cooperative compared to its binding in the absence (Figure 4). For these reasons, the ‘hook effect’ does not apply and does not come into play, at least in the case of molecular glues operating via reversible noncovalent interactions (for an exception to this, see template-assisted or targeted covalent glue degraders that have been found to form covalent adducts in trans with the E3 ligase DCAF16 following binary engagement with their respective target protein Brd4 or Brd9).^4,66^

**Figure 4:**
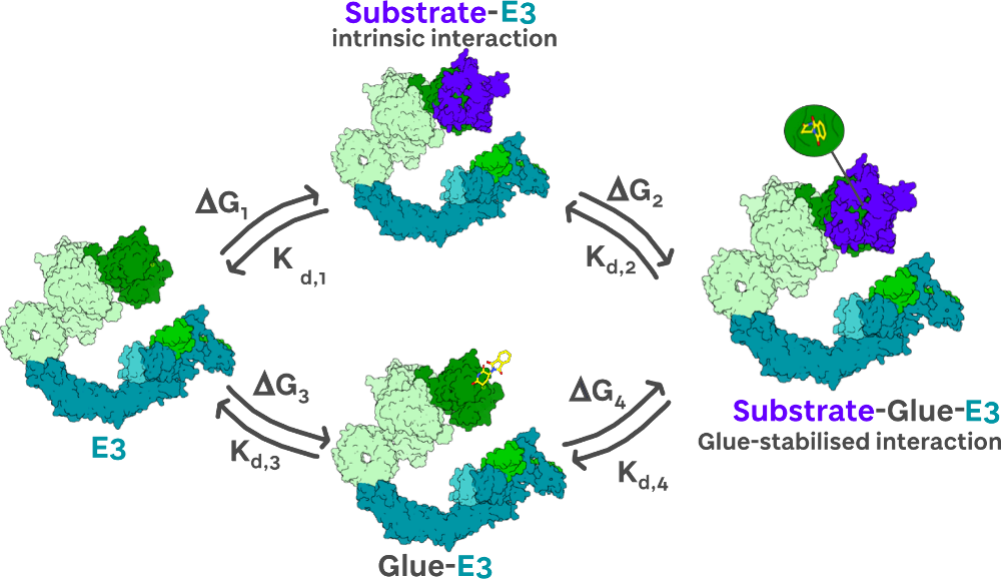
Molecular glue-mediated ternary complex formation. The three-body equilibrium model describing substrate-E3 protein–protein interaction, which upon positive ternary complex cooperativity can be stabilized by a molecular glue. The scenario where the molecular glue binds at binary level to the substrate first, before forming the substrate-glue-E3 ternary complex, is also possible.

For PROTACs, molecular glues, and other noncanonical noncovalent degraders, a faster *k*_on_ and slower *k*_off_ (corresponding to a lower overall K_d_), and a longer half-life *t*_1/2_ of the complex correlate with increased ubiquitination and more efficacious degradation.^57–63^

Unlike ternary complex formation, ubiquitination has been sparsely studied in the context of TPD but constitutes a crucial step for target degradation. While total ubiquitination rate is usually measured, ubiquitination can be separated into two processes: monoubiquitination, which is considered the slower rate-limiting step, followed by more rapid polyubiquitin chain extension.^67,68^ Recently introduced parameters for quantifying the conversion from unmodified to ubiquitinated Brd4^BD2^ are U*b*_max_, the maximum conversion from unmodified to ubiquitinated substrate at a given time point, and UbC_50_, the half-maximal concentration for conversion to ubiquitinated substrate.^69^ A high U*b*_max_ value at >90% and a low nanomolar UbC_50_ value would indicate that the targeted substrate is being ubiquitinated readily and rapidly at high turnover, driving efficient degradation. To be noted, ubiquitination alone may be insufficient for proteasomal degradation. This is because the type of ubiquitin chain linkage must be conducive to recognition by the proteasome to trigger its degradation activity. Moreover, certain ubiquitination sites can induce structural destabilization allowing the targeted protein to be unraveled for degradation at faster rates.^70,71^ However, to date, for the vast majority of cases of degraders hijacking Cullin RING ligase complexes as E3 ligases, it has been found that the greater the ubiquitination level induced by the degrader on the target, the faster, more profound the resulting protein degradation that is observed.^72,73^

Crucially, small molecule degraders require detailed characterization of degradation efficacy. Commonly used parameters are the half-maximal degradation constant DC_50_ and the maximum degradation efficacy value *D*_max_, which are typically obtained by performing concentration-dependent measurements of total protein levels at fixed time-points, using methods such as Western blot or other cellular protein quantification methods and, thus, do not capture the time-dependency of protein degradation (Figure 5 **A** and **B**). More thorough knowledge of how a degrader performs can be obtained by further evaluating the rate of degradation, extent of degradation, and longevity or duration of the degradation. Riching et al. address this by describing additional parameters, illustrated in Figure 5 **C**.^74^

**Figure 5:**
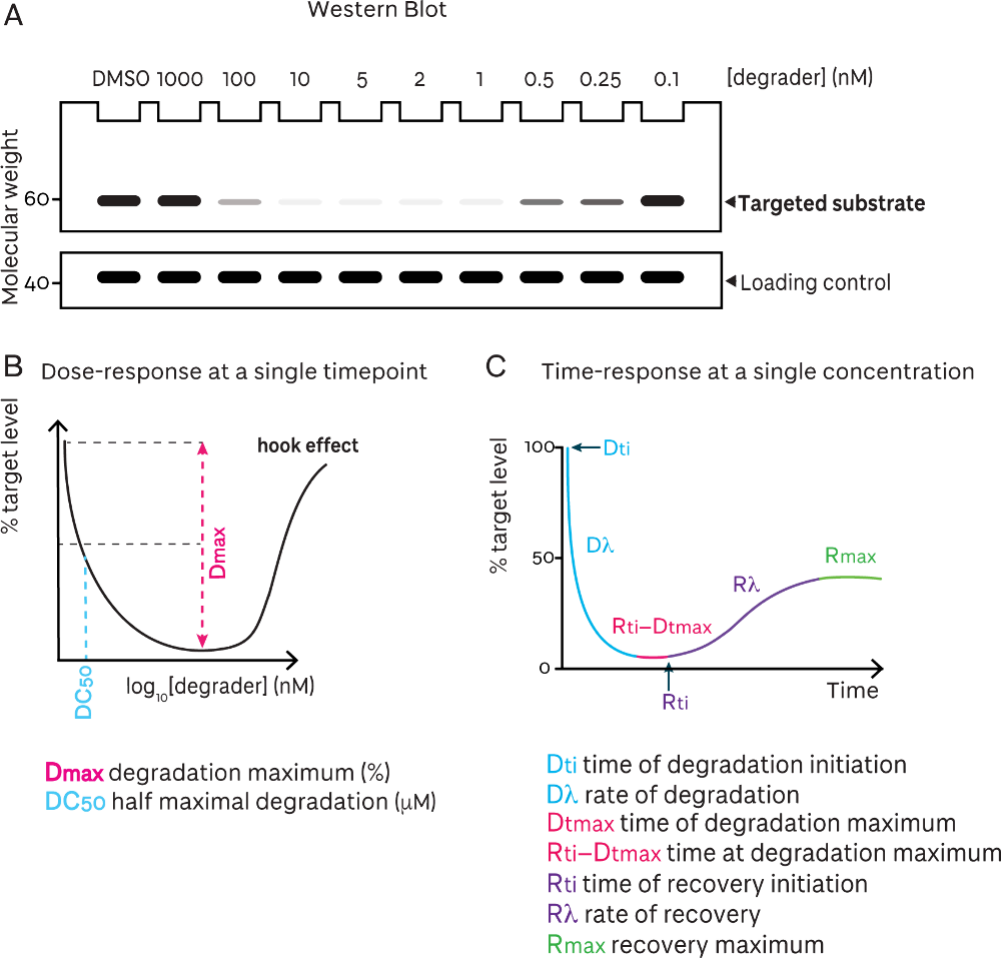
Key read-outs and parameters to evaluate degradation of a targeted protein. **A** Simulated Western blot of a targeted substrate, with a loading control, as a function of degrader concentration. **B** Simulated quantification of the Western blot, representing degradation at a single time point, as a function of degrader concentration. **C** Simulated kinetic degradation, representing degradation at a single concentration, as a function of time *(adaptation reproduced from Riching et al.,* ^74^ *with permission from the Royal Society of Chemistry, Copyright 2022 Royal Society of Chemistry)*.

A further metric which has been proposed includes the ‘degradation score’ Deg_S, which can be calculated from measurements of degrader efficacy combining DC_50_ (in nM), *D*_max_ (in %) observed degradation (in %), degrader concentration (in μM), and incubation time (in hours).^75^ A degrader catalytic rate (DCR) metric has also been proposed, which is based on the concentration of the substrate protein per cell, the degradation rate, and the number of unbound degrader molecules per cell, to better quantify the catalytic activity of a protein degrader.^76^

## 3 Cellular Permeability and Intracellular Availability and Small Molecule Degraders

Consideration of pharmacokinetics is paramount to the performance of a degrader molecule *in vivo*, particularly as physicochemical properties cannot always accurately predict the absorption, distribution, metabolism, and elimination (ADME) profiles for ‘beyond rule-of-five’ compounds. Although several analytical and biomarker-based protocols are used to evaluate distribution, metabolism, and elimination of protein degraders *in vivo* (a review of which is beyond the scope of this paper, but several examples are cited here^77–81^ ), here we outline methods for measuring their cellular permeability and estimating intracellular availability as an important first step to establish biological activities in cellular models (Table 2).

**Table 2:** Overview of Selected Techniques to Predict or Measure Degrader Cellular Permeability

*Method*	*Information*	*Sample preparation*	*Throughput*
*IAM chromatography*	Lipophilicity, polarity, ionizability	N/A	Medium
*Caco-2/MDCK*	Total permeability, efflux	Living cell monolayers; requires serum/BSA	Low
*PAMPA*	Passive diffusion	Lipid-infused filter plate	High
*CAPA*	Intracellular availability	Chloroalkane-tagged degrader; HaloTag cells	Medium
*EPSA (SFC)*	Polar surface area in apolar environments	N/A	High
*NMR*	Intramolecular hydrogen bonds, chameleonicity	N/A	Low

Degrader physicochemical properties are commonly used as an important initial screening method for predicting cellular permeability. These parameters can be obtained through simple analysis of the compound chemical structure (as is the case for the molecular weight, number of carbon atoms, number of rotatable bonds, number of hydrogen bond donors and acceptors, etc.), through computation, or experimentally.

Lipophilicity of a compound is traditionally determined by dissolution in a biphasic system – typically *n*-octanol and water – the two phases are shaken until thermodynamic equilibrium is reached and the concentration of the compound is quantified in each phase. Building on this fundamental methodology but moving away from the low-throughput ‘shake-flask’ setup, more modern chromatographic methods have been developed to improve throughput, material consumption, and sensitivity. In particular, immobilized artificial membrane (IAM) chromatography has gained popularity in recent years as a method to predict drug permeability, yielding readouts on polarity Δlogk_W_^IAM^ based on the measured chromatographic retention time.^46,47^ Two parameters which have been proven in the literature as strong predictors for degrader molecule permeability are the *BR*LogD lipophilicity and Δlogk_W_^IAM^ polarity methods; leveraging high performance liquid chromatography (HPLC) these techniques have very low sample consumption requirements.^46,48^ Other chromatography-based LogD measurement workflows have also been optimized for degraders, such as ChromLogD, which has been shown to work well with highly lipophilic compounds.^82^

Ionization (p*K*_a_) is typically measured experimentally; however, recently a deep neural network (DNN)-based multitask classifier was developed for p*K*_a_ prediction.^83^ This approach was applied to analyze the ionizability of the pan-KRAS degrader ACBI3,^73^ identifying a protonatable amine disfavorable to cellular permeability.^83^ The chromatographic log *k*’80 PLRP-S method provides another alternative to traditional p*K*_a_ determination, and has been shown to accurately predict the propensity of neutral molecules and monoanions to form intramolecular hydrogen bonds (IMHBs).^50^

Jimenez et al. demonstrated that the thermodynamic solubility of PROTACs can be predicted by examining their constituent building blocks, particularly when they share identical terminal functional groups. For example, the increased solubility of (+)-JQ1 carboxylic acid relative to the I-BET726 carboxylic acid drives the enhanced solubility of MZ1 compared to MZP-54.^48,1000^ These findings suggest that strategic selection of high-solubility precursors is a viable guideline for designing more soluble PROTAC molecules.

### 3.1 Direct Measurement of Cellular Permeability

A number of cellular techniques allow for direct measurement of estimated biological permeability and efflux ratio. Cell permeability of drugs is often measured with cell monolayer-based permeability assays such as Caco-2 to determine apparent permeability P_app_ values.^84^ The assay has successfully been applied to PROTACs^85–89^ and molecular glues;^90,91^ however, caution must be exercised particularly for PROTACs and other highly lipophilic compounds. Given the challenges of PROTAC solubility in the assay buffers and nonspecific binding to plates’ wells, low recoveries have been reported.^92,93^ Assay modifications, such as the addition of serum, have been shown to reduce nonspecific binding.^89^ Nevertheless, several studies have reported inaccurate readouts or low precision with large margin of errors, highlighting limited applicability of the Caco-2 protocol to study PROTAC permeability.^92,93^ On the other hand, Caco-2 assays have been used effectively to predict cellular efflux and filter out compounds with high efflux liability prior to further in vivo studies, vide infra and references here.^49,73,86,89^

Further model systems for measuring passive permeability include the Madin-Darby canine kidney (MDCK) and the MDCK-clone Ralph-Russ canine kidney (RRCK) models. These are used less commonly than Caco-2 owing to their lower expression levels of typical drug transporters.^94^ MDCK permeability measurements were performed by Klein et al.; however, several compounds were below the limit of detection in permeation due to their high lipophilicity.^95^ To our knowledge, RRCK has not been applied to small molecule degraders so far.

In the context of small molecule degraders, particularly PROTACs which may be highly lipophilic, these classical methods to evaluate direct cellular permeability may not provide definitive insights without adaptation of experimental methodology.

### 3.2 Fluorescence-Based Methods to Monitor Cellular Influx

Other cellular methodology can include addition of fluorescent tags to monitor cell influx. In the chloroalkane penetration assay (CAPA),^96^ the small molecule degrader is derivatized with a chloroalkane tag.^97^ Foley et al. applied this system to the BET bromodomain degrader MZ1,^97^ where the GFP-labeled haloalkane dehalogenase then forms a covalent bond with chloroalkane-labeled MZ1 present inside the cell, allowing for quantification by fluorescence.^97^ Additionally, this method can be applied to other cellular localizations, and measurement of nuclear penetration can be measured quantitatively.^96^ CAPA can also be used to determine the subcellular efficacy of PROTACs by expressing haloalkane dehalogenase-tagged (HaloTag) target proteins bearing localization signals.^80^ Some limitations exist, in that the compound must be modified with the chloroalkane tail; therefore, the measurements are performed on a modified form and not the true tag-free compound. However no significant decrease in activity was observed on addition of the chloroalkane tag.^97^ Furthermore, given that this reaction is irreversible with the compounds covalently reacting with the HaloTag, this method may not fully account for cellular efflux,^97^ and it may overestimate cell permeability due to compound accumulation aka a ‘sink-effect’.^98^ Intracellular accumulation can be quantified using mass spectrometry of cell lysates, with the caveat that some of the identified molecules could be nonspecifically localized on the cell surface rather than inside the cell.^99^

Extracellular models are also attractive methods, owing to their ease-of-use. For example, Parallel Artificial Membrane Permeability Assay (PAMPA) is a cell-free phospholipid bilayer method for measuring membrane permeability.^100^ This simplified model avoids the use of cells, thereby lowering cost, increasing throughput, but also removing convoluting factors which are present in cell-based models such as active transport or efflux mechanisms.^101^ PAMPA has been shown to have good sensitivity, enabling measurements of PROTACs with expected low permeabilities,^54^ and correlates well with estimates of ‘passive diffusion’ across lipophilic membranes. However, the limit of detection is deemed insufficient to be able to quantify PROTACs which are particularly impermeable and exhibit low recoveries in this assay.^86,99^ To provide further insight, a metric used in combination with PAMPA is the Lipophilic Permeability Efficiency (LPE).^54,95^ Calculated as the difference between the experimental membrane permeability LogD and the calculated octanol/water partition coefficient, LPE measures the extent to which a small molecule achieves membrane permeability relative to its aqueous solubility.^102^ Combining PAMPA and LPE is particularly powerful for larger, more flexible molecules such as PROTACs, which are more prone to intramolecular hydrogen bonding and chameleonic behavior than classical small molecules. For example, Klein et al. illustrated with VH032-based PROTACs and their amide-to-ester analogues that coupling LPE with PAMPA and comparison of LogP can give insight into how the structural arrangement of degrader molecules contributes to compound permeability.^54,95^ Tuning both PAMPA and LPE values in tandem can guide medicinal chemistry approaches in prioritizing a certain scaffold and optimizing for downstream steps (binary binding, ternary complex formation, ubiquitination). It can also inform on whether structural changes such as masking HBDs are required, or if the compound is at risk of poor metabolic stability.

### 3.3 Conformational Ensembles as a Predictor for Cellular Permeability

Beyond computed physicochemical properties and permeability readouts, several studies have focused on the shape, flexibility, and chameleonicity of degraders. This is of particular relevance, as the overall solvent-exposed 3D polar surface area has been shown to correlate with experimentally determined permeability in ‘beyond rule-of-five’ compounds.^103^

Chameleonicity was first measured experimentally with a focus on VHL-recruiting degrader molecules, where studies by Atilaw and Ermondi used 2D-Nuclear Overhauser Effect Spectroscopy (NOESY) NMR to identify proton–proton pairs and determine conformational ensembles of cell-permeable PROTACs. These studies enabled the authors to identify intramolecular and nonclassical hydrogen bonds, p-stacking interactions, and shielding of amide groups from solvent, revealing elongated and polar populations in solutions that mimic extra- and intracellular compartments.^104,105^ Further NMR studies on both VHL- and CRBN-recruiting degraders have rationalized how differences in PROTAC linker flexibility, composition, and folding govern cellular permeability.^106–110^ While NMR has one of the highest requirements in terms of quantity of material, as compared to the other techniques described in this section, a clear benefit is that the material can be retrieved from the NMR tube and reconstituted for further experiments or analysis.

Chameleonic behavior has also been qualitatively estimated by reverse-phase chromatography using mobile phases spanning different scales of polarity, based on the assumption that molecular chameleons adapt their conformation in apolar environments by reducing their polar surface area.^87^ Recently, a supercritical fluid chromatography (SFC)^45^ technique was developed to measure EPSA for degrader molecules, with a focus on high-throughput screening to rank-order human permeability for PROTACs.^111^

Further studies have shown that chameleonic behavior can be estimated from molecular dynamics simulations.^106,112^ Overall, molecular chameleonicity is an emerging but highly relevant molecular feature that should be considered for degrader molecules, particularly for PROTACs.

### 3.4 Methods to Evaluate Small Molecule Degrader Cellular Efflux and Uptake

In recent years, the cellular efflux pumps ABCC1/MRP1 and ABCB1/MDR1 have been shown to act on PROTACs to a significant extent.^113,114^ In fact, by monitoring efflux ratio, Kofink et al. showed that changes to PROTAC linker design, favoring the compound to adopt a more compact 3D conformation, lead to a reduced efflux ratio and therefore much improved overall oral bioavailability.^89^ Furthermore, some PROTACs have been shown to be metabolized in-cell, leading to enhanced clearance and efflux.^79,114^ In a study by Chen et al., rigidification of the linker region in a BTK-targeting PROTAC led to improved metabolic stability over the PEGylic linker analogue, which in turn led to more effective cell growth inhibition. These examples highlight a need to monitor metabolism and efflux as a significant barrier for degrader intracellular availability and bioavailability. Efflux susceptibility can also be measured at a downstream stage, for example, DC_50_-dependence on ABCB1/MDR1 inhibition by zosuquidar or ABCC1 inhibition by reversan, which has been applied in the context of degrader molecules in several studies.^73,109,114,115^ Furthermore, some metabolites might have a similar binding affinity for either the substrate or E3 ligase, potentially competing with the full degrader molecule and impeding the degrader mechanism of action, and can also present further challenges such as toxicity, as is the case, for example, with the metabolite hydroxy-thalidomide, which still binds to CRBN and acts as a molecular glue degrader itself.^79,92,116,117^ Considerable progress has been made over recent years to develop computational tools to predict drug metabolites, including the application of deep learning strategies.^118–121^ Computational predictions could prove beneficial in the early stages of projects and should be coupled with further drug metabolism and cellular efflux studies using complementary experimental methods.

In addition to efflux transporters, CD36 has recently been highlighted as an uptake transporter implicated in PROTACs entering the cell.^122^ The authors identified CD36 by performing chemical pull-down and global proteomics. While the authors performed CD36 knockout to verify PROTAC influx dependency, new CD36 inhibitors could be helpful to rapidly check for CD36 dependency without the need for knockout cell lines.^123^

Given these recent findings, future strategies for PROTAC design could include optimizing binding to CD36 to enhance cellular uptake, as has been shown by Wang et al.,^122^ while also minimizing binding to efflux transporters. Identifying the involvement of these transporters could provide valuable insight for PROTAC targets which present challenges in terms of tissue activity, oral bioavailability, or brain-blood barrier penetration. It is possible that these challenges could have some degree of dependency on the varying expression levels and activities of such transporters in different cell types and tissues.

## 4 Small Molecule Degrader-Mediated Binary Binding and Ternary Complex Formation

### 4.1 Biochemical Methods to Evaluate Degrader-Induced Ternary Complex Size and Oligomerization

The critical step in the TPD mechanism of action is the formation of a ternary complex between the E3 ligase, the degrader, and the target protein. Several label-free biochemical techniques can determine whether ternary complexes have formed and evaluate mono- and multimeric protein ensembles, for example, size-exclusion chromatography (SEC), electrophoretic mobility shift assay (EMSA), mass photometry, and native mass spectrometry (Figure 6). These methods inform on a change in molecular weight consistent with an increased state of hetero- or homo- oligomerization.

**Figure 6:**
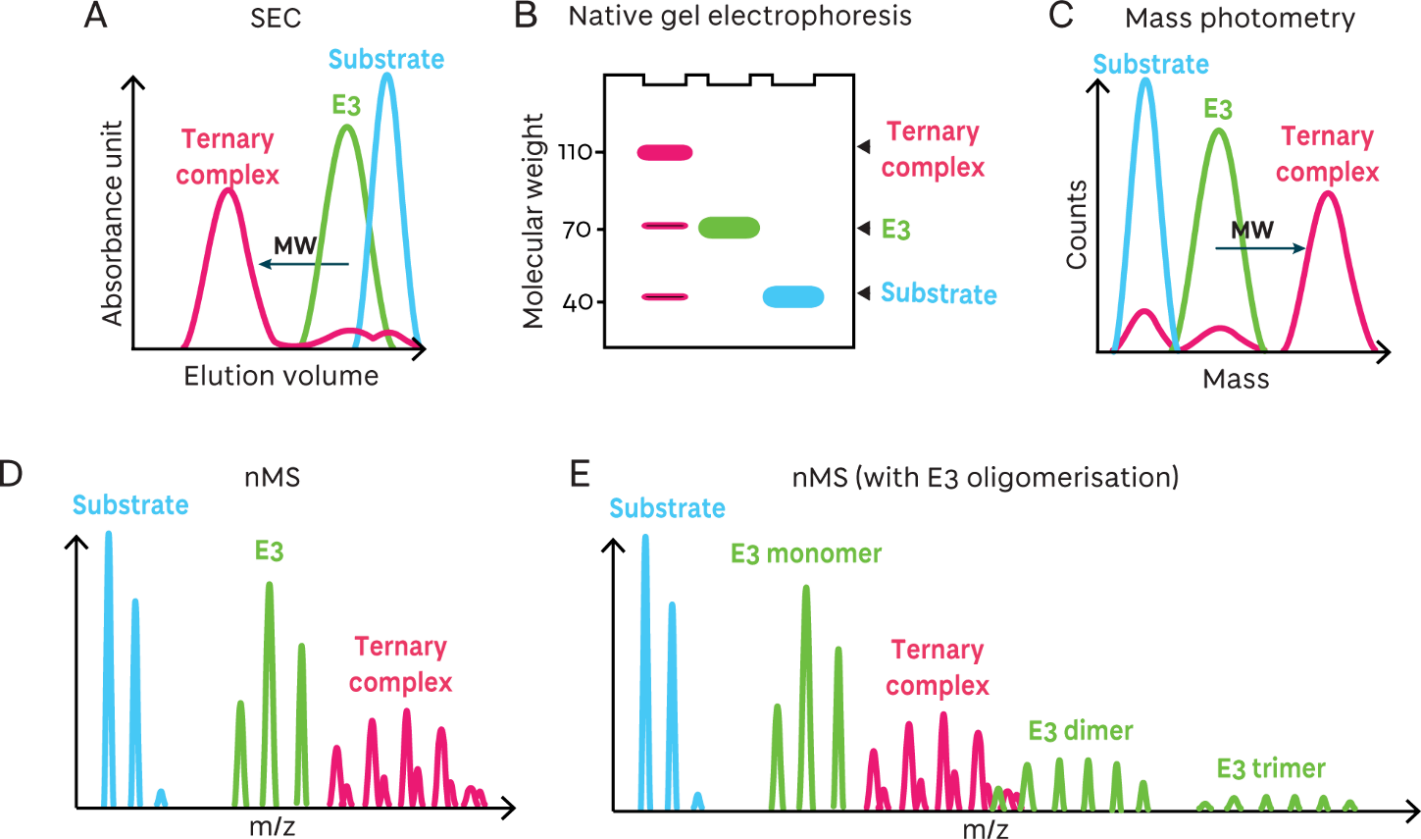
Overview of biochemical methods for monitoring degrader-induced ternary complex binding. **A** Schematic of a SEC chromatogram showing a shift in elution volume on ternary complex formation. **B** Schematic of a stained native gel showing an upward shift in molecular weight on ternary complex formation. **C** Schematic of a Gaussian-fitted mass photometry histogram showing a rightward shift in molecular size on ternary complex formation. **D** Schematic of a native mass spectrum showing a rightward shift in mass-to-charge ratio (*m*/*z*) on ternary complex formation. **E** Schematic of a native mass spectrum showing a rightward shift in mass-to-charge ratio (*m*/*z*) on ternary complex formation as well as a rightward shift in mass-to-charge ratio on protein oligomerization.

SEC analysis reveals a shift in elution volume of the injected complex correlating with an increase in molecular weight with the ternary complex formation (effect illustrated in Figure 6 **A**). This method has been applied to a number of systems, for example, to study homoPROTAC-induced dimerization of VHL-EloC-EloB complex (VCB),^124^ heteroPROTAC-dependent VCB/BET bromodomain complex formation,^125^ and DCAF16/Brd4^tandem^ complexes with IBG1 and IBG3.^3^ Performed on a fast protein liquid chromatography (FPLC) purification system, analysis by SEC has the added advantage of producing purified ternary complex which can be used for onward structural analysis, for example, by X-ray crystallography and cryo-EM.^3,126^ Quantification of the ratio of species present can be performed by integrating the areas under the curves, and an approximate molecular weight can be determined by reading the elution volume in relation to a standard curve obtained from species with a known molecular weight. However, the resolution can be limited depending on the quality of the SEC column, flow rate, and concentration of the injected species. Moreover, proteins and complexes of different shapes and especially those that deviate significantly from globularity, e.g., because they include large unstructured regions, are retained differently and lead to inaccurate prediction of MW from this approach alone. The throughput of this assay depends largely on the type of SEC column used, with smaller analytical-scale columns capable of running a single sample in as little as 7 min. If further accuracy on mass-readout is required, SEC can also be coupled with multiangle light scattering (MALS), which measures the intensity of scattered light on elution from the SEC column (this combined method is commonly referred to as SEC-MALS).

The EMSA technique is another facile way to observe a shift in mass upon ternary complex formation (effect illustrated in Figure 6 **B**), and it has the added benefit of being economical in cost, requiring only gel electrophoresis apparatus and reagents. Diehl et al. developed a native gel electrophoresis method to evaluate the ability of homoPROTACs to dimerize recombinant VHL protein under nondenaturing conditions and showed a correlation between the percentage of ternary complex formation detected and the degradation levels of pVHL30 observed by Western blot.^124^ Quantification of the bands can give an indication of the ratio of the different species present; however, like SEC this technique is limited in resolution and sensitivity. This assay is typically lower throughput, requiring a nondenaturing gel to be cast, and protein samples can take 1–2 h to obtain the desired resolution. Advantageously, multiple samples can be resolved on the same gel simultaneously.

For mass photometry, protein concentrations for measurements must be low (in the range of 100 pM-100 nM) to allow detection of single protein particles adsorbing to the glass slide. A study by Schwalm et al. used mass photometry to show 98–99% ternary complex formation between 25 nM VCB and 25 nM WDR5 in the presence of a high concentration of 10 μM degraders MS67 and AD122 (effect illustrated in Figure 6 **C**).^62^ Similarly, Crowe et al. showed complete ternary complex formation between NEDD8-CRL2^VHL^, MZ1, and BRD4^BD2^ at a 1:1.2:1.2 mol equiv ratio. Mass photometry has also proven useful in studying degrader-dependent protein multimerization. Huang et al. reveal DCAF15-DDB1-DDA1 dimers and trimers in the absence of degrader at 20 nM protein concentration and a shift toward monomers and dimers in the presence of 20 nM protein and 200 nM molecular glue E7820.^127^ This method provides a good overview of the sample dispersion and the species present with very little sample consumption, and readouts can be obtained sequentially in as little as 1 min. However, the state-of-the-art in mass photometry detection is limited to protein species above 40 kDa. At very low protein concentrations, a large excess of a small molecule can sometimes be required to render the ternary complex analytically tractable.^128^ A workaround method that can help with ternary complex identification involves preincubating the protein and small molecule species at micromolar concentration and rapidly diluting them to nanomolar concentration directly before the measurement.

Native mass spectrometry (nMS) allows the detection of intact proteins in their native states, preserving their folding and interactions, and therefore can be used to study the formation of protein complexes with small molecules and other proteins. Beveridge et al.^129^ applied the nMS method to detect PROTAC-bound species for the system comprising VCB, the BET bromodomains Brd4^BD1^, Brd4^BD2^, and Brd3^BD2^, and the PROTACs MZ1 ^5^ and AT1.^16^ They confirmed that nMS revealed a higher fraction of ternary complex formation with Brd4^BD2^ than Brd4^BD1^ and Brd3^BD2^, with even better selectivity with AT1 than MZ1,^129^ in full agreement with previously published SPR^59^ and ITC^16^ data. This method has since been applied to other PROTAC and molecular glue systems including VCB/BET bromodomains,^130,131,1001^ 14–3–3/ERα/p53/LRRK2 proteins,^132^ 14–3–3/Pin1 proteins,^133^ CRBN/GSPT1 proteins,^134^ VHL/ACBI3/KRAS,^1001^ and also to identify the oligomerization of DCAF15-DDA1-DDB1 with/without RBM39 and/or E7820.^127,134^ Shifts in mass-to-charge ratio on ternary complex formation and protein oligomerization are illustrated in Figure 6 **D** and **E**. Depending on the sensitivity of the instrument, nMS is well-suited for high affinity pM-low μM range of dissociation constants.

Overall, these biochemical techniques are valuable for visualizing which ensembles of species are present. In particular, SEC, EMSA, and MP are limited in resolution and are less likely to capture more transient interactions. nMS can achieve higher mass resolution and sensitivity. Here, detection of higher μM-mM interactions is feasible; however, optimization in terms of instrument sensitivity, gentle ionization conditions, higher protein concentrations, and stabilizing buffer conditions may be required.

### 4.2 Biophysical Methods to Evaluate Degrader Binary and Ternary Complexes *In Vitro*

Biophysical assays of varying throughput are effectively used to produce the quantitative data required for assessing the equilibria of binary and ternary complex formation. This information has proven crucial for degrader development. Specifically, potent and fast degraders act at low concentrations to form ternary complexes of high thermodynamic stability and kinetic longevity.^16,57–63^ Therefore, evaluating and optimizing this step of the mode of action is critical for degrader optimization. Biophysical methods to evaluate degrader complexes can be categorized into proximity-based, competition-based (Figure 7), or direct binding, and are summarized in Table 1.

**Figure 7:**
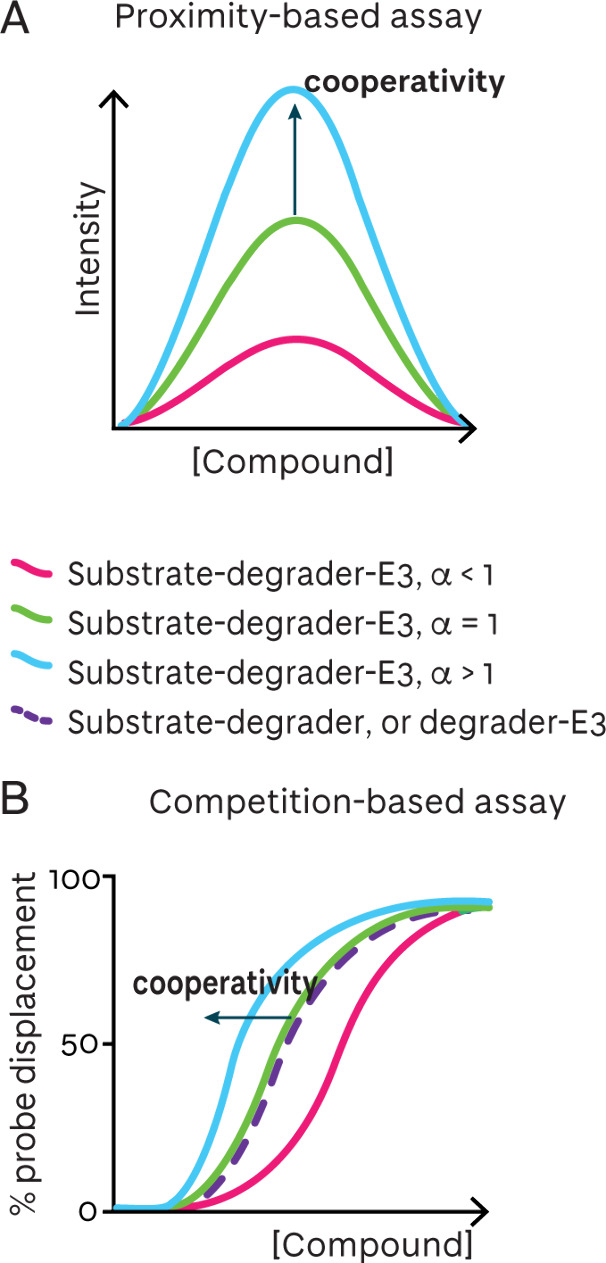
Typical curve shapes in proximity-based and competition-based degrader-induced ternary complex binding. **A** Proximity-based assays produce a bell-shaped curve, where the amplitude is increased with increasing positive ternary complex cooperativity and decreases with negative cooperativity. **B** Competition-based assays produce a sigmoidal curve, where the curve is left-shifted with increasing positive ternary complex cooperativity and right-shifted with negative cooperativity.

#### 4.2.1 Proximity-Based Assays

Amplified luminescent proximity homogeneous assay-linked immunosorbent assay (AlphaLISA)^135^ and time-resolved Förster resonance energy transfer (TR-FRET) are proximity-based methods that follow a similar mechanism for ternary complex detection (Figure 8). In these methods, both the E3 ligase and the target protein are labeled with donor and acceptor species. Upon proximity induced by a degrader, light excitation at the wavelength of the donor species results in energy transfer from the donor to the acceptor species, generating an emission signal. For AlphaLISA, energy transfer is from a ‘donor’ bead which, on excitation, produces short-lived singlet oxygen species with a diffusion radius of 200 nm.^136^ On ternary complex formation, the ‘acceptor’ fluorophore is excited by the singlet oxygen, producing a readout at the wavelength of the acceptor species (Figure 8 **A**). By contrast, in TR-FRET, energy transfer is from a donor fluorescent FRET pair dye to its acceptor FRET pair dye (Figure 8 **B**). The acceptor species is another fluorophore that will produce an emission at a wavelength distinct from the donor if it is located within the Förster radius (2–9 nm for most FRET fluorophore pairs).^137^ Increases in these emission signals can be detected and interpreted as increased ternary complex formation. The AlphaLISA proximity assay was first established with degraders by Gadd et al. to screen a library of MZ1-derivative PROTACs against VCB and BET bromodomains, and the authors observed an increase in signal amplitude with increased ternary complex cooperativity (this effect is illustrated in Figure 7 **A**).^16^ Despite the advantages of the assay, the authors additionally highlighted that AlphaLISA data should be interpreted with caution owing to the multiplicity of binding sites and relative linkage orientation of components immobilized to the donor and acceptor beads.^16^ Since then, the assay has been expanded to other E3s,^11,138^ other target proteins,^57,139–143^ and E3 ligase dimerization with homoPROTACs,^144^ and it has been extended to cell lysate systems to study small molecule degraders.^142^

**Figure 8:**
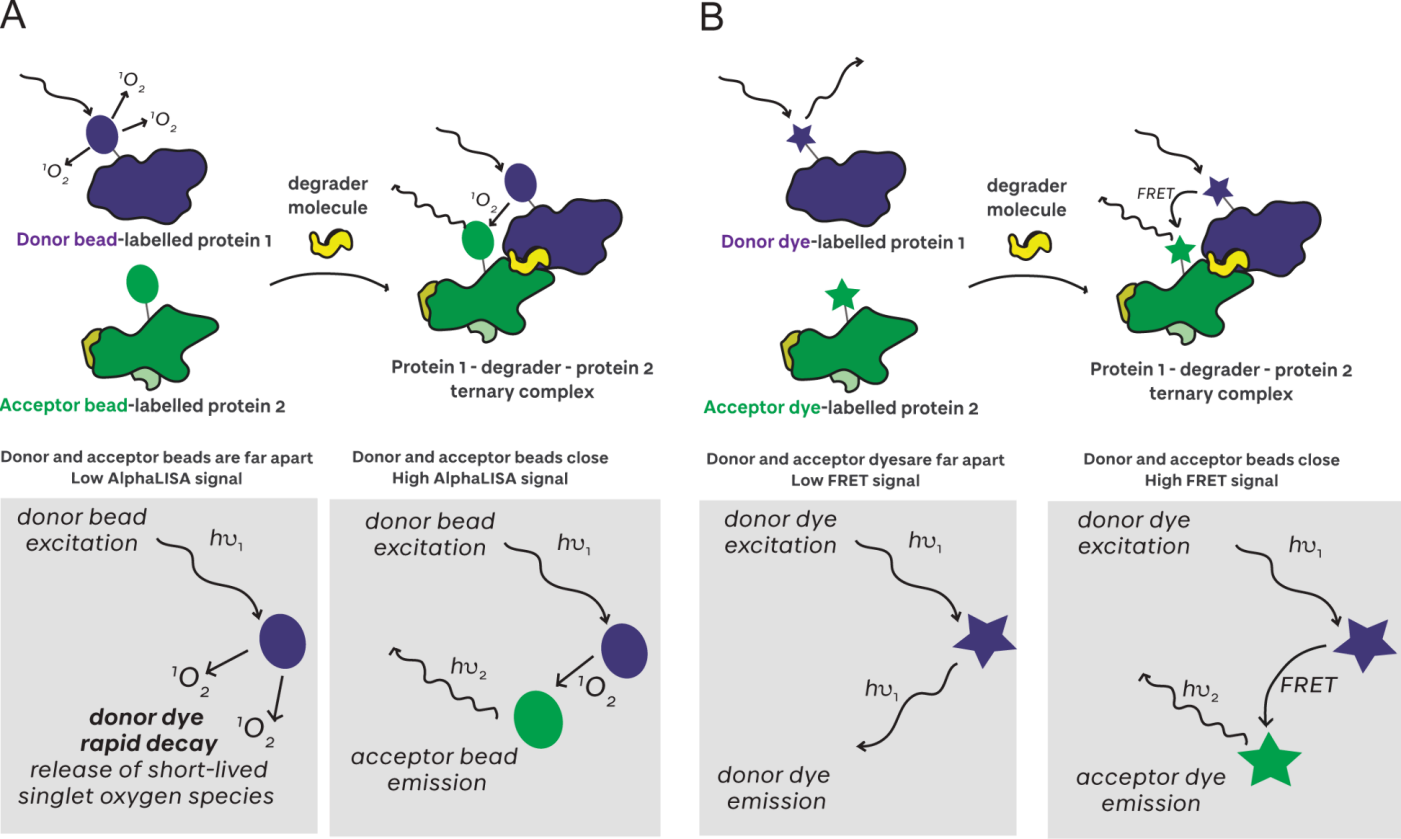
AlphaLISA and TR-FRET for proximity-based ternary complex measurements. **A** In AlphaLISA experiments, excitation of a donor bead generates a singlet oxygen species. Upon ternary complex formation, proximity between the donor and acceptor beads allows for the acceptor to be excited by the single oxygen species and emission at the wavelength of the acceptor dye. **B** In TR-FRET experiments, excitation of a donor dye generates emission at a certain wavelength. Upon ternary complex formation, proximity between the donor and acceptor dyes allows for energy transfer and emission at the wavelength of the acceptor dye.

TR-FRET, which requires a shorter distance between donor and acceptor species, has been applied to a similar scope of E3/target protein systems. Recent examples include a recent DCAF1-Brd9 PROTAC,^10^ the intramolecular bivalent glue (IBG1) recruiting Brd4^tandem^ and DCAF16,^3^ molecular glues degrading cyclin K through the CDK12/DDB1 interface,^145^ *neo*-substrate proteins recruited to CRBN by molecular glue degraders,^146^ and a VHL-based molecular glue degrader for CDO1.^147^ A study from Przytulski et al. aiming to compare AlphaLISA with TR-FRET for degrader ternary complex analysis showed that despite the different assay principles, both assays yielded comparable ternary complex responses in the studied test system.^148^ While these assays are amenable to a variety of systems and can have good throughput and low sample consumption, the complex multiparameter binding equilibria that yield the characteristic bell-shaped curves for PROTACs mean that fitting and extracting binding affinities can be challenging, particularly because of the characteristic “hook effect”. Despite requiring functionalization of both proteins of interest, good throughput can be achieved by performing these assays in a plate-based format, and protein concentrations are low, in the nanomolar range, making AlphaLISA and TR-FRET highly sensitive methods useful for screening degrader libraries for ternary complex formation.

#### 4.2.2 Competition-Based Assays

Another category of experimental methods for evaluating degrader binary interactions and degrader-induced ternary complex formation is competition assays. Here, a probe usually composed of a well-known binary ligand functionalized with a reporter species generates a baseline signal while bound to its partner protein, and is displaced upon complex formation (Figure 7 **B**).

AlphaLISA (Figure 9 **A**) and TR-FRET (Figure 9 **B**) can both be used in a competition setup to assess binary binding; for example, Guenette and Potts developed a high-throughput AlphaLISA assay to identify new ligands for E3 ligases by titrating unlabeled degron peptides to displace biotinylated peptides bound to E3 ligases.^149^ These methods can also be applied to measure ternary complex formation through probe displacement; for example, AlphaLISA assays by Imaide and Hsia both use a biotinylated JQ1 probe displaced on compound titration (SIM1 and IBG1, respectively) to characterize binary complexes and ternary complex formation between Brd4^tandem^ and VCB and DCAF16 in the presence of degrader,^3,150^ and CRBN engagement has been quantified using a CRBN-binding probe.^76^ Similarly, TR-FRET has been applied to evaluate binary BET bromodomain binding^151^ and binary CRBN binding of STAT5 degraders.^152^ While competition AlphaLISA and TR-FRET can both be applied to measure binary and ternary binding, these assay set-ups require a well-established labeled ‘probe’ ligand, and ternary complex formation parameters cannot be read directly, meaning that they have to be extracted by comparing ‘protein A’-degrader binding performed in the absence versus in the presence of ‘protein B’, which must assume binding saturation with protein B.

**Figure 9:**
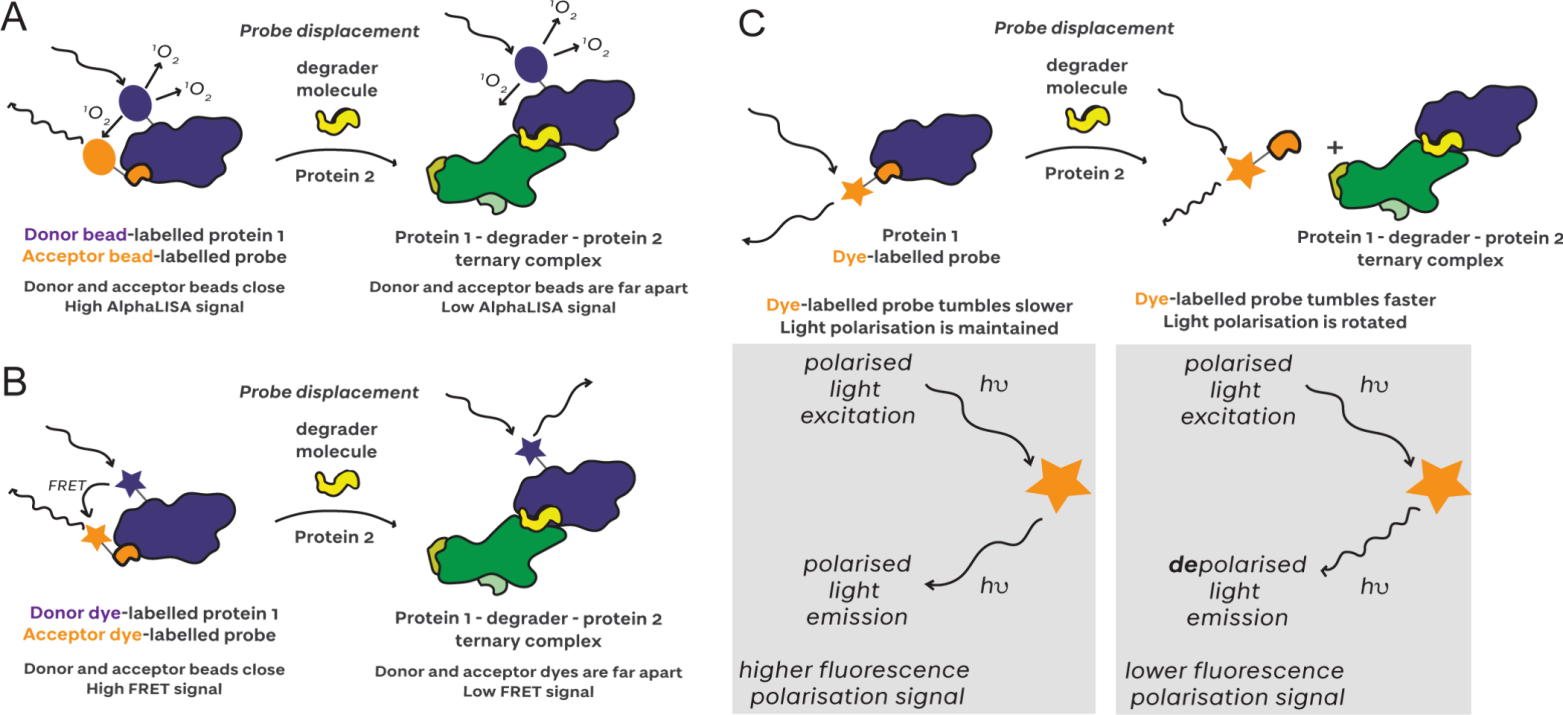
Competition-based ternary complex measurements with **A** AlphaLISA, **B** TR-FRET, and **C** FP.

Similarly, Lumit has been applied in a competition-based system. Using a GST-tagged BRD4 and a biotinylated BET tracer, Crumming et al. are able to detect a luminescent signal using LgBit- and SmBit-conjugated streptavidin and anti-GST Lumit reagents. As with AlphaLISA and TR-FRET, the binding characteristics of PROTACs are revealed through competition with the BET tracer.^153^

Competition fluorescence polarization (FP) can also be used to evaluate binary and ternary binding^154^ and, advantageously, does not require tagging of the E3 or target protein (Figure 9 **C**). Instead, the small molecule probe which binds to the E3 or target protein is functionalized with a fluorescent dye (often fluorescein-based, or similar). Incubation of the protein and probe in solution results in a baseline signal which is attenuated on probe displacement with a stronger-binding compound or compound-protein pair. Competition FP proved key in developing nonpeptidic small molecules for binding VHL competitively to the substrate HIF-1α derived peptides: these FP screening campaigns led to the key ligands used for VHL-recruiting PROTACs.^155,156^ The same method has recently been followed to enable ligand development for another E3 ligase, FEM1C.^157^ Competition FP is also one of the most commonly used competition-based assays for evaluating degrader ternary complexes and cooperativities. Competition assays are not affected by the hook effect, allowing for full compound titrations series and quantification of binding. A study by Zoppi et al. compared the PROTAC-induced displacement of fluorescent HIF1-α peptides and found their PROTACs showed negative cooperativity with a rightward shift in the IC_50_ curve in the presence of the Brd9 bromodomain.^141^ In contrast, degrader ternary complexes which are positively cooperative display a leftward shift relative to the binary binding curve or relative to noncooperative systems (illustrated in Figure 7 **B**), as was the case for a different bromodomain target, SMARCA2/4, with VCB and PROTACs leading to the development of ACBI1,^142^ as well as for the different BET bromodomains engaging MZ1:VHL.^59^ Because of their sensitivities and throughput, FP ternary complex competition assays have been widely incorporated into degrader characterization and degrader screening campaigns.^158^

All four of the competition-based assays described use very low concentrations and low volumes of ‘protein 1’ and probe (nanomolar range). However, for ternary complex measurements, the third binding partner ‘protein 2’ is typically present at much higher concentrations, in the range of 10–25 μM. Nevertheless, these assay setups are amenable to plate-based screening. While these present powerful techniques, competition-based assays present the same drawback in that they require the development and validation of a well-characterized fluorescently labeled probe ligand.

#### 4.2.3 Biosensor-Based Direct Binding Methods

Biosensor-based biophysical methods can be highly informative for profiling molecular binding over traditional solution-based strategies. The immobilization of protein species on the surface of a chip can result in an increase in local concentration of protein, allowing for real-time and sensitive detection of molecular interactions. As a result of this enhanced sensitivity, kinetic rate constants *k*_on_ and *k*_off_ can be measured and K_d_ and *t*_1/2_ values calculated.

The development of surface plasmon resonance (SPR) for PROTAC-recruited ternary complex characterization constituted a landmark in the TPD field’s understanding of degrader-mediated ternary complex formation dynamics. First applied by Roy et al.,^59^ the authors designed a VHL-EloB­(AviTag)-EloC construct for EloB-biotinylation. The resulting biotinylated VCB was immobilized onto a streptavidin-coated SPR chip, and the kinetics of binary VCB-PROTAC interactions were first measured. To measure ternary complex formation, the BET bromodomain protein and PROTAC were preincubated, before being flowed over the VCB on the SPR chip (Figure 10 **A**).^59^ Following this first study, SPR is now routinely used for characterizing degraders by immobilizing either the E3 or the target protein,^10,11,159–163^ and it has also been extended to screening approaches,^164^ including screening of off-rates from unpurified reaction products.^165^ While initially applied mainly to VHL-recruiting PROTACs, with the recent development of smaller and more soluble CRBN protein constructs such as CRBN-midi,^161,166^ SPR with CRBN immobilization has now also been successfully performed to study CRBN binding ligand and ternary complexes.^167^

**Figure 10:**
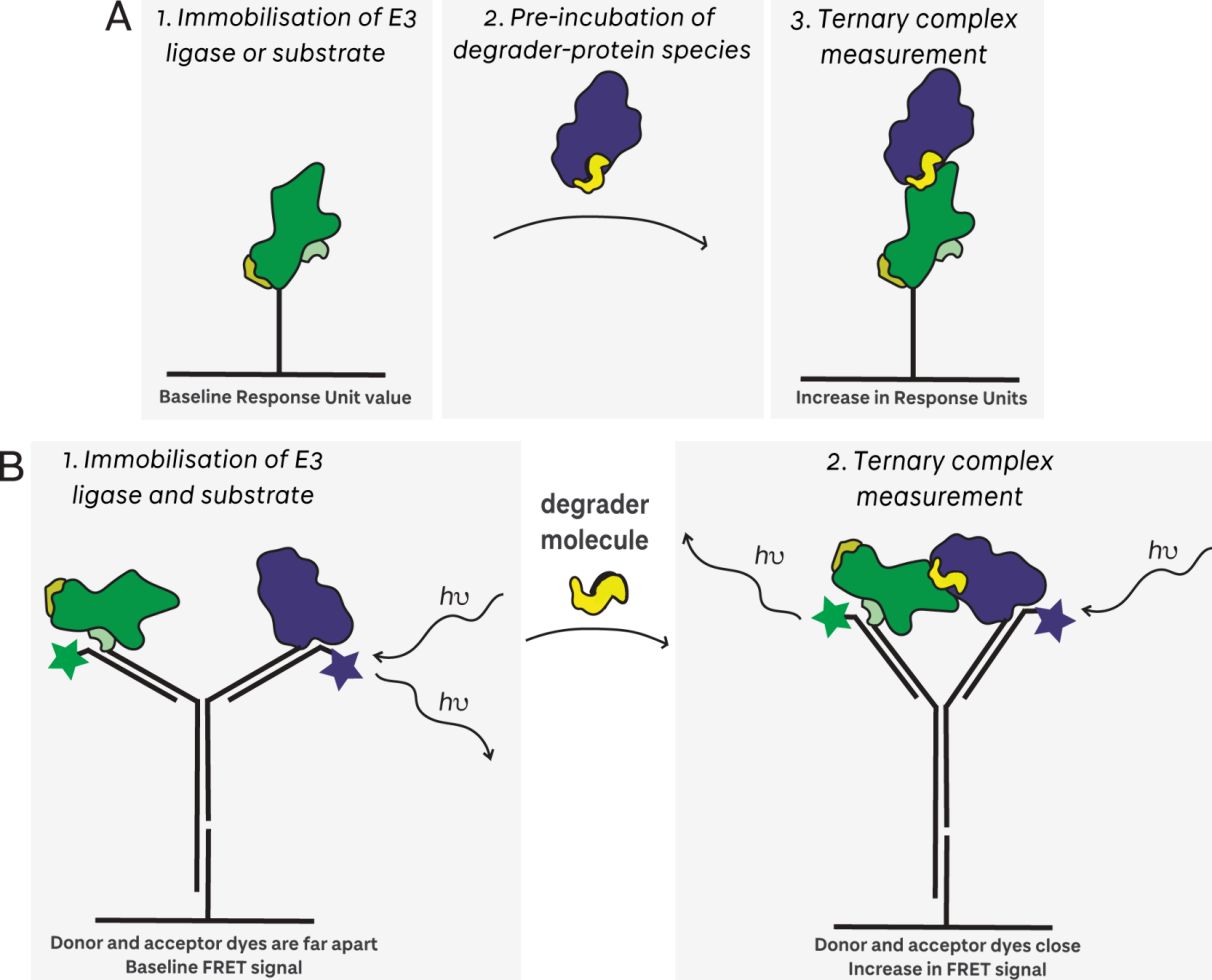
Immobilization setups used in degrader ternary complex measurements using biosensor methods. **A** In SPR and BLI, a single protein species is typically immobilized. **B** In heliX Y-structure assays, both protein species are typically immobilized.

Biolayer interferometry (BLI) has also been successfully applied to study degraders. While most studies use BLI to study PROTACs and molecular glues in binary binding,^148,168–172^ Schiemer et al. developed a BLI method to measure ternary complex formation.^173^ The authors immobilized biotin-cIAP onto the streptavidin-coated BLI biosensor tip and dipped this into a PROTAC solution. After briefly washing, the binary cIAP-PROTAC biosensor tip was then dipped into BTK solution to obtain ternary complex *k*_on_, *k*_off_, and K_d_.

An additional method, using the heliX biosensor, has recently been developed to quantify degrader binary and ternary complexes. In their study, Ponzo et al.^174^ immobilized the E3 ligase proteins (VHL-EloB-EloC or DDB1-CRBN) and the BET bromodomain proteins on the surface of the biochip through their lysine residues via NHS ester coupling with single-stranded DNA. Binary degrader association and dissociation is measured through quenching of a fluorescent dye present on the DNA strand in proximity to the immobilized protein. For ternary complex measurements, a DNA ‘Y-structure’ was functionalized with both the E3 ligase and the BET bromodomain proteins (Figure 10 **B**). Using this dual-immobilization, degrader-mediated ternary complex *k*_on_ and *k*_off_ rates were measured by monitoring FRET from a donor dye to an acceptor dye.^174^

A recently published biosensor-based method with potential to study small molecule degrader ternary complex formation is escape-time stereometry (ETs). Introduced by Zhu et al., this single-molecule method relies on protein species diffusing through biochip-immobilized entropic traps, providing an average escape time (*t*_*esc*_) correlating with the molecule’s hydrodynamic radius. Given that this is a single-molecule approach, sample consumption is low, and the high level of sensitivity would offer the opportunity to capture weak interactions.^175^ To our knowledge, this technique has not yet been applied to TPD.

Given the relevance of calculating degrader-induced ternary complex half-lives, with more long-lived ternary complexes correlating with increased rate and amplitude of protein degradation, biosensor methods allowing measurements of off-rates are powerful for degrader optimization campaigns. While biochip immobilization is advantageous from this perspective, complications relating to nonspecific biochip surface binding can convolute results; therefore, some optimization of measurement conditions is often necessary to ensure data fit well to anticipated simple 1:1 binding models.

#### 4.2.4 Other Direct Binding Methods

Isothermal titration calorimetry (ITC) is a direct-binding method which played a major role in the development of binary VHL binding ligands^156,1002^ and, later, in assessing the binary binding of VHL-recruiting PROTACs, and it was then extended to study CRBN-recruiting ligands and degraders.^5,176^ ITC is label-free and measures exothermic or endothermic changes following titration of ligand into a protein solution, or vice versa, to evaluate binary binding. To measure ternary complex formation, Gadd et al. developed a method to first titrate the substrate BET bromodomains into a solution of PROTAC to measure binary binding and ensure there would be no remaining excess PROTAC, then titrate VCB into the saturated substrate-PROTAC solution to measure ternary complex formation.^16^ Despite being relatively low-throughput, ITC has become a widely used technique in the TPD field, as it is the only technique capable of measuring binding thermodynamics and relative stoichiometry. Exemplary recent applications of ITC to study degraders ternary complexes include studying VHL homoPROTAC dimerizers,^144^ VHL-based Brd9 ^141^ and SMARCA2/4 ^142^ PROTACs, CRBN-recruiting CDK12-cyclin K molecular glues,^13^ and IBGs binding to tandem BET bromodomains and DCAF16.^3^

Nuclear magnetic resonance (NMR) spectroscopy, both protein-observed where the protein is isotope-labeled or ligand-observed where the small molecule is monitored, can be applied to observe the small molecule ligand binding to proteins and even to measure the dissociation constant of the complex. NMR has been extensively used to study binding of small molecules to E3 ligases, for example, in the early work to develop VHL ligands, using both ligand-observed^156,177,178^ and protein-observed NMR,^179^ including more recently by the Fesik laboratory.^180^ However, few reports have used this approach in the context of small molecule degraders and especially their ternary complexes. De Castro et al. introduced a ligand-observed NMR experiment to estimate the cooperativity parameter α for a series of degraders.^181,182^ A ^19^F-labeled ligand for the VHL E3 ligase was developed^182,183^ and was then used in a competition-based assay. By titrating PROTACs against VHL and thus displacing the ^19^F-probe, and also titrating BET bromodomain complex against VHL, a comparison between binary and ternary displacement allowed the authors to estimate positive and negative cooperativity from the IC_50_ curves.^181^ Ramachandran et al. further extended this approach to measure competitive binding of peptides and small-molecules binding to the phospho-tyrosine binding pocket of the SH2 domain containing E3 ligase SOCS2, by displacement of a bespoke-designed fluorinated ‘spy’ probe molecule, obtaining comparable K_d_ values to orthogonal SPR direct binding measurement.^184^

Thermal denaturation assays (TDA), otherwise known as thermal shift assays (TSA), measure protein thermal denaturation, monitored through two methods: fluorescence or aggregation.^185^ Differential scanning fluorimetry (DSF) uses an environmentally sensitive fluorescent dye, such as SYPRO Orange, which binds to hydrophobic regions that become exposed as a protein unfolds or denatures upon increasing temperature, causing an increase in fluorescent signal.^186,187^ In the native, folded state of the protein, these hydrophobic residues are typically buried within the protein’s core, and as a result the dye is quenched in aqueous solutions. Typically, protein thermal stabilization occurs upon ligand binding, which increases the protein’s melting point (*T*_m_). DSF has been applied to some degrader binary systems.^188–191^ DSF is also amenable to high-throughput screening; for instance, Lucas et al. applied this method to a library of 1200 fragments screened against VCB.^178^ NanoDSF (nDSF) is another method which can inform on protein thermal stabilization on ligand binding; however, the readout of nDSF comes from the change in intrinsic fluorescence from tryptophan in the protein. nDSF has been applied to a degrader study to monitor binary binding.^125^ Dynamic light scattering (DLS) is another type of TSA, which is label-free; here, a thermal gradient is applied causing protein unfolding and aggregation, which can be monitored by measuring the intensity of scattered light. Thus far, the use of DLS for degrader discovery, development, or characterization has not been reported. While these techniques do present advantageous aspects, the denaturation temperature (*T*_m_ shift) cannot always be readily transformed into binding affinities, and the high denaturation temperatures used to rank ligand affinities does not necessarily reflect the ligand performance at physiological temperatures.^192^

Microscale thermophoresis (MST) detects the changes in spatial distribution of fluorescently labeled proteins when a thermal gradient is applied. Ligand binding is expected to induce changes in the local environment of the fluorescent dye caused by the ligand itself or changes in the conformation of the protein on ligand binding. MST was introduced to study PROTAC-induced binary binding and ternary complex formation between VHL and BET bromodomains.^193^ Since then, the technique been extended to other binary^194–197^ and ternary systems such as VHL and BCL-XL ternary complex^198^ as well as VHL/EGFR ternary complexes.^199^ Maiwald et al. extended the MST methodology to a competition-based assay using fluorescently labeled reporter ligands and measured displacement from CRBN and VHL with binary binding ligands.^197,200^

Other less widely used methods with potential to study small molecule degrader binary binding and ternary binding include spectral shift assay (SpS),^201^ where a case study has recently been published,^202^ and flow-induced dispersion analysis (FIDA) monitoring the fluorescently labeled protein through a flow capillary to monitor hydrodynamic radius (*R*_h_), thus far unpublished (Table 3).

**Table 3:** Overview of *In Vitro* Biochemical and Biophysical Techniques to Measure Degrader Binary and Ternary Binding and Influence the Chemical Design of Degrader Molecules

*Method*	*Binding type*	*Information*	*Labeling/immobilization*	*Protein consumption*	*Throughput*	*Detectable K*_*d*_ *range*
*SEC*	Ternary	MW	Label-free	5–100 μM, 5–500 μL	7–35 min/sample	pM-μM
*Native gel*	Ternary	MW	Label-free	1–10 μM, 5–10 μL	1–2 h/10–12 samples	pM-μM
*MP*	Ternary	MW	Label-free	0.1–100 nM, 10 μL	1–2 min/sample	pM-nM
*nMS*	Ternary	*m*/*z*	Label-free	5–10 μM, 10 μL	5–30 min/sample	pM-μM
*AlphaLISA (proximity)*	Ternary	IC_50_	Label E3 & POI with donor and acceptor beads	5–500 nM, 10–25 μL	2–3 h/plate	pM-nM
*TR-FRET (proximity)*	Ternary	IC_50_	Label E3 & POI with FRET-pair fluorescent dyes	5–500 nM, 10–25 μL	1–2 h/plate	pM-nM
*AlphaLISA (competition)*	Binary & ternary	IC_50_, K_d_	Label E3 or POI & probe with donor and acceptor beads	4 nM-25 μM, 10–25 μL	2–3 h/plate	pM up to K_d_ of probe
*TR-FRET (competition)*	Binary & ternary	IC_50_, K_d_	Label E3 or POI & probe with FRET-pair fluorescent dyes	2–40 nM, 10–25 μL	1–4 h/plate	pM up to K_d_ of probe
*FP*	Binary & ternary	IC_50_, K_d_	Label ligand probe with fluorescent dye	5 nM-25 μM, 10–25 μL	5 min/plate	pM up to K_d_ of probe
*ITC*	Binary & ternary	K_d_, ΔH, ΔS, N	Label-free	20–200 μM, > 200 μL	40 min/sample	nM-5 mM
*SPR*	Binary & ternary	K_d_, *k*_on_, *k*_off_	Immobilise E3 or POI with affinity tag	100 nM-25 μM, 150 μL	20–40 min/sample	pM-2 mM
*BLI*	Binary & ternary	K_d_, *k*_on_, *k*_off_	Immobilise E3 or POI with affinity tag	5 nM-100 μM, 200 μL	20–40 min/sample	pM-mM
*heliX*	Binary & ternary	K_d_, *k*_on_, *k*_off_	Label E3 & POI with ssDNA	500 nM, 2 μL	5–10 min/sample	pM-μM
*Protein-observed NMR*	Binary & ternary	IC_50_, K_i_	Isotope label E3 or POI	50–200 μM, > 120 μL	1–12h/sample	pM-mM
*Ligand-observed NMR*	Binary & ternary	IC_50_, K_i_	Isotope label ligand	50–200 μM, > 120 μL	5–120 min/sample	pM-mM
*DSF*	Binary	*T*_m_	Label-free	5–25 μM, 10–20 μL	15–30 min/sample	pM-mM
*nDSF*	Binary	*T*_m_	Label-free	5 μM, 10–20 μL	75 min/sample	pM-mM
*DLS*	Unpublished	*R*_h_	Label-free	unpublished	unpublished	unpublished
*MST*	Binary & ternary	F_norm_	Label E3 or POI with fluorescent dye	10 nM-10 μM, 10 μL	1 min/sample	pM-mM
*SpS*	Binary & ternary	spectral shift	Label E3 or POI with fluorescent dye	5–50 nM, 20 μL	1 min/sample	pM-mM
*FIDA*	Ternary	*R*_h_	Label E3 or POI with fluorescent dye	5–100 nM, 5 μL	unpublished	pM-mM

### 4.3 Biophysical Methods to Identify Degrader Binary and Ternary Complexes in Cells

Given the complexity of the intracellular environment, binary binding and ternary complex formation *in vitro* may not fully reflect a degrader’s efficacy *in cellulo* and *in vivo*. Cellular target engagement is dependent on several factors, including compound cell permeability, export/efflux, sequestration, and target protein compartmentalization. Moreover, engagement of the endogenous full-length target protein inside the cell may differ from that measured *in vitro* with recombinant purified protein constructs. Therefore, cellular biophysical assays are useful to complement *in vitro* measurements.

An opportunity to measure the extent of binary binding in a cellular system was presented by Riching et al. with the NanoLuc-VHL fusion assay, which monitors nano-bioluminescent resonance energy transfer (NanoBRET) between a NanoLuc-protein fusion and a HaloTag-labeled reporter ligand.^58^ Initially developed to monitor MZ1-derivative PROTACs engagement with VHL in HEK293 cells (Figure 11),^58^ this assay has been expanded to multiple systems and is today used widely.^10,72,150^ ^,^ ^203–206^ By performing these binary engagement assays both with whole cells and permeabilized cells and comparing the results side by side, the assay can provide an indirect readout of degrader cell permeability.^205^ This approach also has been extended to monitor ternary complex formation, where the labeled species are now the target protein and the E3 ligase.^58,63,140^ ^,^ ^150,207–208^

**Figure 11:**
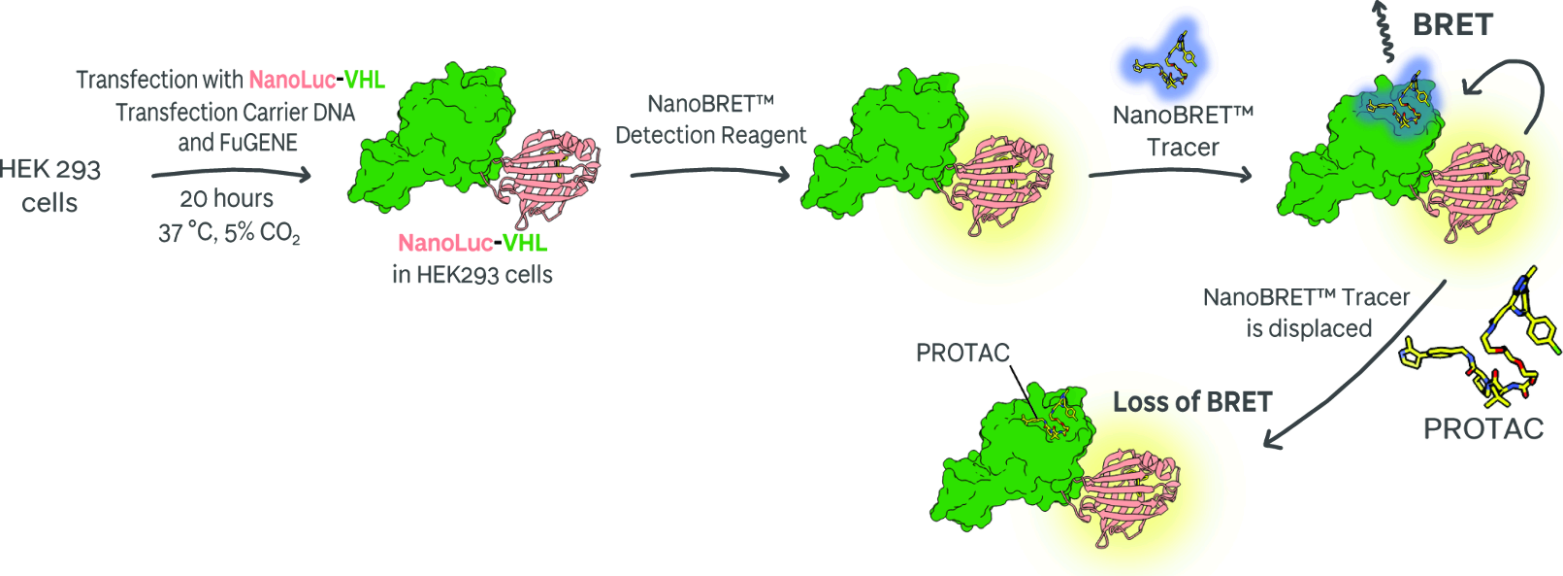
Schematic representing the general assay workflow for measuring binary target engagement between PROTACs and a NanoLuc-labeled VHL in live cells. The chemical structure of the fluorescent NanoBRET Tracer species is proprietary, and the compound shown here is for illustrative purposes only.

Further fluorescence-based methods based on protein complementarity have been engineered to examine intracellular interactions; for example, biomolecular fluorescence complementation (BiFC)^209^ was recently applied by Lei et al. to detect levels of AKT-PROTAC-CRBN ternary complex formation.^1003^ Here, AKT and CRBN were both tagged with fragments of EGFP or luciferase, and ternary complex formation induced association of the two protein fragments, yielding a fluorescent signal.

TSA can also be applied in a cellular context with cellular TSA (CETSA). The assay was first applied to small molecule degraders by Winter et al. to monitor cellular engagement of Brd4 by dBET6 and dBET1 in CRBN-deficient cells.^6^ Smith et al. also performed this assay, with VHL-recruiting PROTACs targeting p38α.^143^ Here, the authors treated cells with the PROTAC and applied a temperature gradient to denature and precipitate unbound proteins; in contrast, ligand-bound proteins remained soluble and could be quantified by Western blot following cell lysis, thereby quantifying binary binding.^143^ Quantitative mass spectrometry-based proteomics can be combined with CETSA to profile a degrader’s interactions in whole cells, providing a way to assess target specificity.^210^

A caveat to these cellular engagement assays is that there is the possibility of the target engagement results to be confounded with depletion of the protein induced by the degrader compounds. Thus, care must be taken to circumvent and control for engagement versus depletion, for example, performing short treatments and cotreatment with neddylation and proteasome inhibitors such as MLN-4924 and MG132.^210^

The use of fluorescent PPi (fluoPPi) methods combined with live cell confocal microscopy visualization offers a strategy to monitor ternary complex formation in cellular regions through imaging of the green photostable mNeonGreen protein. This assay has been applied to degrader-mediated CRBN ternary complexes with Brd4, FKPB12^F36 V^, and CBP/EP300.^211,212^ Separation of phases-based protein interaction reporter (SPPIER) is a similar assay to fluoPPi but, instead, uses the nanoluciferase (NanoLuc) protein as a fluorescent reporter. SPPIER is applied to both CRBN and VHL-based systems; however, it does not have the same level of spatial resolution as fluoPPi.^213^

A method which has proven useful for confirming ternary complex formation is co-immunoprecipitation. Here, one of the protein binding partners can be overexpressed in cells with a high-affinity tag such as FLAG, HA, or streptavidin. After treatment with a compound, pull-down followed by a Western blot can determine if the ternary complex is present. While this technique is useful to confirm ternary complex formation in cells for known binding partners, for example, with VHL/p38α ^143,214^ and DCAF16/FKBP12,^215^ the approach can also be extended to systems where one of the binding partners is unknown and combined with mass spectrometry analysis to identify the other components of the complex.^216–218^

### 4.4 Methods to Structurally Evaluate Degrader Binary and Ternary Complexes

Several structural biology techniques have been applied thus far to study degrader binary and ternary binding and characterize their molecular feature at the atomic level, summarized in Table 4.

**Table 4:** Overview of Structural Techniques to Study Degrader Binary and Ternary Complexes

*Method*	*Information*	*Sample preparation*	*Material consumption*
*X-ray crystallography*	High-resolution information	Crystallization	Medium
*Single particle cryo-EM*	Variable resolution on complexes >40 kDa, sample heterogeneity	Application to cryo-EM grid and plunge-freezing or spraying	Low
*Protein-observed NMR*	Residues involved in binding, of complexes <30 kDa	E3 or POI protein isotope labeling	High
*HDX-MS*	Solvent-exposed residues	Exchange with deuterium, quenching and digestion	Low
*IM-MS*	Complex conformations and assemblies	N/A	Low
*SAXS*	Complex size and shape	N/A	Low

To date, X-ray crystallography has been the main method to obtain structural information on degrader binary binding and ternary complex formation. This method requires a good amount of high-purity protein and has some drawbacks; for example, complexes may not crystallize, or crystals may not diffract to good enough resolution, or capture the complex in just one of many conformations accessible in solution, sometimes biased by non-native crystal contacts. Despite these limitations, X-ray crystallography is a powerful technique and probably the best available for obtaining high-resolution information on the protein binding site and binding mode, as well as to reveal the bioactive conformation of the small-molecule bound, which can be used for further structure-guided drug design. Indeed, a series of studies from Ciulli, Crews, and colleagues relied on cocrystal structures to guide the design of the first small-molecule ligands for VHL.^17,155,156^ The development of these VHL-ligands through structure-guided design allowed for the creation of the first nonpeptidic VHL-recruiting PROTACs.^5,9,219^ The protein X-ray crystallography study by Gadd et al. featured the first reported PROTAC ternary complex crystal structure, between VCB (VHL-ElonginC-ElonginB) as substrate-adaptor components of the E3 ligase, the PROTAC MZ1, and Brd4^BD2^ as the target protein.^16^ Through this crystal structure, the authors captured extensive MZ1-mediated PPis between VHL and Brd4^BD2^, also showing the PROTAC folding into itself to allow for specific intermolecular interactions in the ternary complex. Using biophysical methods to characterize ternary complexes, Gadd et al. revealed the specificity of MZ1 for Brd4^BD2^ over other BET bromodomains, and the structure solved rationalized how MZ1 forms the most stable and cooperative ternary complex with Brd4^BD2^, which also explained its degradation preference for Brd4^BD2^ and allowed for the structure-based rational design of a novel PROTAC named AT1 with improved Brd4^BD2^ specificity. Structure-guided design based on the crystal structure by Gadd et al. has yielded further series of VHL-recruiting PROTACs, some of which have also been characterized in their ternary complexes.^220,221^ Since, many more VHL-PROTAC ternary structures have been published, and the approach has also been extended to CRBN. Since the identification of cereblon as the E3 ligase recruited by thalidomide,^2^ X-ray crystallography has been applied to understand how thalidomide and lenalidomide recruit CRBN in a binary binding complex.^18,19^ These efforts spearheaded by the Thomä and Cathers laboratories then led to X-ray crystal structures of CRBN in a ternary complex with their molecular glue and their *neo*-substrates.^65,218,222,223^ The development of truncated cereblon constructs has yielded further binary and ternary X-ray crystallographic degrader structures.^161,166^ While X-ray crystallography is a crucial technique for providing high-resolution information, an inherent drawback to crystallization is that any information on conformational heterogeneity is lost. Thus, in-solution techniques can give further insight into the complexity of degrader-protein systems.

Single-particle cryo-electron microscopy (cryo-EM) has been used to solve degrader-protein complexes. Cryo-EM for degrader structural characterization was initially established in 2019 with DCAF15-DDB1 complexes,^224,225^ then extended to the CRBN E3 ligase substrate receptor by Watson et al. with a series of binary molecular glue-CRBN structures.^226^ Further binary CRBN binders have been characterized with cryo-EM by Lejava et al.,^227^ and degrader-mediated ternary complexes have also been solved using cryo-EM, such as cereblon-molecular glues in complex with Ikaros and SD40.^172^ Cryo-EM of ternary complexes has allowed for key insights into new degrader binding modalities; for example, the DCAF16-IBG1-Brd4^tandem^ complex gave insights into an intramolecular bivalent glue and guided the design of improved picomolar-potent degraders,^3^ and the ternary structure between DCAF16-Compound 1-SMARCA2 showed that Compound 1 recruits SMARCA2 to DCAF16 through a covalent bond and in a structurally distinct manner relative to BRD4 recruited by IBG1.^228^ While resolutions on cryo-EM structures are largely reduced and heavily localized compared to X-ray crystallography, thus limiting information on small molecules which are typically not well-resolved by cryo-EM, the recent ongoing ‘resolution revolution’ with significant progress in terms of cryo-EM hardware and data processing promises to enhance the utility of this technique by achieving higher resolutions. Many more degrader ternary complex structures have now achieved overall resolutions of below 4 Å.^3,4,73,229–234^

Most structural studies on PROTAC ternary complexes disclosed to date have been restricted to the E3 ligase substrate receptors, but cryo-EM has been used to visualize the fully assembled multisubunit catalytically active E3 ligase complex NEDD8-CRL2^VHL^ in complex with MZ1 and the recruited non-native substrate Brd4^BD2^.^69,235^ With the recent development of deep learning neural network models and algorithms, such as 3DFlex,^236^ cryoDRGN,^237^ 3D variability analysis (3DVA),^238^ and e2gmm,^239^ among others, cryo-EM is now a powerful method to explore heterogeneity and flexibility. For example, for binary complexes, these methods have contributed to mapping cereblon open/closed conformations^226^ and highlighting flexibility in cereblon ternary complexes.^172^ Crowe et al. applied 3DVA to resolve continuous flexibility of the full degrader-induced Brd4^BD2^-MZ1-NEDD8-CRL2^VHL^-UBE2R1-Ub complex, revealing the range of motion available to the Cul2 scaffold protein and informing on the possible interfaces between the recruited *neo-*substrate protein and the E2 catalytic site.^69^

Protein-observed NMR spectroscopy is an in-solution technique which can also yield atomic-level structural resolution; however, the approach has not been widely extended to study small molecule degrader-mediated structures. Protein-observed NMR has been used by Schiemer et al. to study degrader-mediated ternary complexes between BTK and cIAP1. The authors used ^1^H/^15^N-HSQC to qualify the binary and ternary complexes and showed that there were no stable PPis forming between BTK and cIAP1, thus demonstrating that the degrader-induced ternary complexes were noncooperative.^173^ Protein-observed NMR studies in TPD are limited, likely due to the size limitation of 30 kDa due to line broadening and spectral crowding in conventional NMR experiments and the complexity of spectral assignment; however, a more modern methodology such as TROSY and/or protein perdeuteriation could push the resolution limit.^240,241^ Despite its low-throughput and high sample consumption and the need for well-assigned spectra, protein-observed NMR holds potential for studying degrader-mediated binary and ternary complexes and would allow the capture of transient species in solution.

Mass spectrometry constitutes a useful approach to structurally evaluate degrader binary and ternary complexes. Hydrogen–deuterium exchange mass spectrometry (HDX-MS) can inform on the solvent exposure of amide hydrogens, by exchanging with deuterium followed by quenching, digestion, and peptide analysis to determine which residues have increased in mass due to deuterium uptake. Conformational changes induced by binary degrader binding can be resolved by HDX-MS; for example, Watson et al. showed reduced solvent exposure on cereblon consistent with a transition from ‘open’ to ‘closed’ states on molecular glue binding.^226^ Ternary complex formation can also be studied with HDX-MS; for instance, Dixon et al. highlight residues on VHL and SMARCA2/4 which form PPis when the two proteins are engaged in ternary complexes with ACBI1 and other PROTACs,^242^ and Eron et al. applied HDX-MS to study CRBN/Brd4 ternary complexes.^243^ Ion-mobility mass spectrometry (IM-MS) can inform on native protein conformations and interactions present in solution. Song et al. use IM-MS to reveal that the MZ1-mediated ternary complex between VCB and Brd4^BD2^ can adopt a distribution of conformations.^131^

Small-angle X-ray scattering (SAXS) can also be applied to TPD by informing on complex size and shape. This method has been applied to study binary binding, where Kroupova et al. showed their novel CRBN construct was capable of transitioning from ‘open’ to ‘closed’ conformations on binary binding with iberdomide, mezigdomide, and lenalidomide, consistent with insights from Watson et al. using full-length CRBN-DDB1 complex.^161^ Ternary complex formation was also shown with SAXS by Dixon et al. for the VHL-ACBI1-SMARCA2/4 system.^242^

In recent years, computational methods have been developed to predict and model degrader ternary complexes. Methods such as HADDOCK^244^ and Rosetta^20^ offer potential for *in silico* screening prior to small molecule degrader chemical synthesis and biological evaluation. Computational methods have also been developed to model PROTAC-mediated ternary complex formation;^245–250^ several of these have been reviewed elsewhere.^35,251^

## 5 Methods to Evaluate Target Protein Ubiquitination

### 5.1 Biophysical Methods to Monitor Ubiquitination Levels *In Vitro* and in Cells

In addition to E3-degrader-substrate ternary complex formation, engagement of the ubiquitin-loaded E2 followed by discharge of ubiquitin onto a *neo*-substrate lysine and building a polyubiquitin chain must occur to trigger polyubiquitinated substrate recognition, unfolding and degradation.

Initially, ubiquitination was sparsely measured in the context of TPD, commonly employing end-point assays which involve stopping the reaction at a given time. These experiments can be performed *in vitro* using purified recombinant proteins. Cellular ubiquitination levels can also be evaluated, requiring preincubation of cells with proteasome inhibitor MG132 to rescue ubiquitinated target protein followed by co-immunoprecipitation or affinity tag pull-down and Western blot to identify ubiquitination through visualization of a distinctive ‘polyubiquitin ladder’ effect.^64,68,143^ ^,^ ^252–254^ Visualization of degrader-targeted substrates has also been explored through radiolabeling.^9^ While this is highly sensitive, such methods are disfavored due to radiation hazards. Ubiquitination kinetics have been evaluated in a study by Vieux et al.; however, this method also relies on time-points and immunoblotting.^255^ While effective for confirming ubiquitination of the targeted protein, these experimental approaches are only semiquantitative and do not allow for continuous readouts, thereby limiting extrapolation of real-time kinetic data and kinetic parameters for the enzyme-catalyzed reaction.

The development of fluorescent small molecules with enhanced sensitivity and associated instrumentation has offered the opportunity for more sensitive and time-dependent ubiquitination event monitoring.^256^ Crowe and coauthors developed an Alexa Fluor 647 directly- and covalently labeled Brd4^BD2^ protein, which, following *in vitro* ubiquitination with Alexa Fluor 488-labeled ubiquitin and resolution of the protein species by SDS-PAGE, allowed for quantification of U*b*_max_ and UbC_50_ parameters at given time points.^69^ Outside of the context of TPD, fluorescence polarization of ubiquitin has been measured,^257,258^ as well as FRET and single molecule FRET (smFRET) between a labeled ubiquitin species and a labeled substrate.^259–261^ FRET has also been applied in a study by Schroder et al. to measure PROTAC-dependent ubiquitination of BTK.^10^

Another tool of interest for ubiquitination monitoring is tandem-repeated ubiquitin-binding entities (TUBEs). Introduced by Hjerpe et al., the TUBE technology is based on a series of four ubiquitin-associated domains (UBAs) capable of recognizing tetra-ubiquitin.^262^ With this tool, polyubiquitinated proteins can be pulled out from recombinant systems and cell extracts for analysis. Of note, TUBEs can protect its bound polyubiquitin chains from DUBs and proteasomal degradation; however, proteasome inhibitors are usually still used in these assays. This approach can be used in a magnetic bead TUBE pull-down approach in conjunction with Western blotting against the targeted protein and mass spectrometry analysis.^263^ The commercialized ‘UbiTest’ assay kit extends this approach to a plate-based format, which Gross et al. applied to study BET protein ubiquitination mediated by the dBET PROTACs.^264^ While this methodology avoids the need for an antibody against the targeted protein, its signal could be attenuated by background noise caused by other polyubiquitin species, and the method does not allow for kinetics to be resolved. TUBEs are also used in the commercial plate-based ‘UbiQuant’ assay where the AlphaLISA bead-labeled TUBE and an antibody against the targeted protein yield an increase in signal when a polyubiquitinated target protein. An example of Brd3 ubiquitination by dBET1 has been published.^263^ TUBEs bearing a fluorescent label such as TAMRA or fluorescein have also been explored;^263^ thus far, no experiments have been reported to use these with small molecule degraders.

A study by Riching et al. first measured degrader-induced ubiquitination kinetics in a cellular context, and from a series of bioluminescence-based cellular assays the authors drew correlations between cellular ternary complex formation and increased levels of cellular target ubiquitination, suggesting that productive ubiquitination stems from productive ternary complex formation.^58^ This NanoBRET ubiquitination assay has since been successfully applied to a number of other systems.^3,72,141^ ^,^ ^150,265^

### 5.2 Structural and Mechanistic Characterization of Degrader-Targeted Protein *Ubiquitinability*

In addition to kinetic insights informing on rates of ubiquitination, methods that inform on the structural and mechanistic detail of how degraders influence productive and selective target protein ubiquitination are highly desirable to develop effective degraders.

Up until recently, investigations of this nature have remained sparse and primarily featured computational modeling. Degraders recruiting the E3 CRL2^VHL^ have been characterized through modeling and MS; for example, Gadd et al. modeled CRL2^VHL^ in complex with MZ1, Brd4^BD2^, UBE2D, and ubiquitin and identified lysine ubiquitination sites on BET bromodomains modified in an MZ1-dependent *in vitro* assay, with the residue K346 shown to be modified on Brd4^BD2^ being in proximity to the E2 active site. Khan et al. also used MS and found K87 as the only lysine on BCL-X_L_ with their VHL-recruiting DT2216 PROTAC. More recently, there have been several studies focused on CRL4^CRBN^-recruiting systems. In a study by Bai et al., the authors made predictions on cyclin-dependent kinase (CDK) ubiquitination efficiency by a number of cereblon-recruiting PROTACs using Rosetta modeling to visualize the full CRL4 catalytic machinery, and they also performed MS to map ubiquitinated lysine sites.^265^ Molecular dynamics simulations were performed by Wu et al. to understand how surface lysines on Brd4^BD1^ are arranged relative to the CRL4A-recruited E2 catalytic pocket.^266^ A study by Zhao mathematically modeled ternary complex dynamics of VHL-, CRBN-, and BIRC2-recruiting systems and highlighted ubiquitination zones presenting lysines close to the E2 catalytic sites on targeted *neo*-substrates.^267^

Recently, cryo-EM has provided an experimental method to obtain structural detail of ubiquitination. This technique has been applied in studies by Crowe et al. and Li et al. to solve structures of Brd4^BD2^ in complex with MZ1 and CRL2^VHL^, using UBE2R1 and UBE2R2, respectively.^69,235^ Using UBE2R1, Crowe and coauthors developed a flexible cross-linked species for use in cryo-EM and highlighted K456 at optimal positioning for nucleophilic attack. Following *in vitro* ubiquitination of recombinant Brd4^BD2^, the authors used MS and mapped K-GG modification sites to reveal a ‘light face’ of Brd4^BD2^ which can be ubiquitinated by CRL2^VHL^/UBE2R1, with K456 being the only lysine showing evidence of ubiquitination and having the highest intensity of modification at the time points investigated.^69^ While obtaining high-resolution cryo-EM structures of multisubunit, flexible, and dynamic complexes is challenging, the approach proves to be powerful for visualizing which orientation is adopted by the *neo-*substrate, how many lysines are accessible for ubiquitination, and ultimately understanding how ubiquitinable the recruited *neo-*substrate is in the system by the studied degrader.

‘Ubiquitinomics’, i.e., global ubiquitin mapping of the proteome, can provide insights into endogenous target protein lysine modification. This technique has been applied in studies by Dixon et al. and Cantley et al. to map ubiquitinated lysines on SMARCA2 and SMARCA2/4, respectively,^242,268^ and by Crowe et al. to study ubiquitinable lysines on BET protein following cellular treatment with MZ1.^69^ The approach has the added strength of revealing potential off-target ubiquitination which may lead to off-target degradation or alternative undesired downstream effects, knowledge which is important when advancing degrader-development campaigns particularly with regards to safety.

Various computational methods have been developed to predict ubiquitination based on flanking protein sequence segments, using databases of experimentally validated ubiquitination sites.^269^ These methods, which usually combine a mixture of parameters including, but not limited to, amino acid composition (AAC), amino acid pairwise composition (AAPS), positional weighted matrix (PWM), position-specific scoring matrix (PSSM), and solvent-accessible surface area (SASA) as well as machine learning operations, include ESA-UbiSite, DeepUbi,^270^ UbiComb,^271^ and others.^272–274^ To date these methods have not been applied in TPD studies in the literature.

Overall, these studies rely mostly on modeling and prediction, or on challenging cryo-EM campaigns, coupled with *in vitro* or cellular proteomics mass spectrometry; nevertheless, there have been correlations drawn between the number of accessible lysines on the target protein and the degradability of the target.^242,275^

### 5.3 Deciphering Degrader-Mediated Ubiquitin Chain Assemblies

In addition to overall structure and lysine accessibility to the E2 catalytic site, the target protein must be ubiquitinated with a polyubiquitin tag, constituted of K48 and/or K11 linkages, that are conducive to degradation because they allow recognition and processing by the 26S proteasome.^276^ Mass spectrometry is the most explored method to characterize ubiquitin chain linkages. For example, using a cellular proteomics approach Ottis et al. established that CRL2^VHL^ and CRL4^CRBN^ can mediate K11 and K48 linkages on their degrader-recruited *neo*-substrates.^277^ Ways to determine the exact topology of ubiquitin chain formation, distinguishing between single polyubiquitin chain and multiple mono- or polyubiquitin chains and elucidating linear versus branched chains, are under development. A study by Kaiho-Soma et al. revealed how TRIP12 associates with CRL2^VHL^ to facilitate K29/K48-branched chain formation on Brd4 and how TRIP12 recruitment accelerates Brd4 and Brd2 degradation.^278^ The authors applied Lb^pro^*, an engineered viral protease,^279^ to decode the branched ubiquitin linkage architectures.^278^ Outside of TPD, Meyer et al. applied MS methods to determine that K11/K48 chains are more easily recognized by p97 and the proteasome, suggesting that chain branching could be favorable for degradation.^280^ Deubiquitinases such as isopeptidase T, which has been shown to break down polyubiquitin chains, can be applied to generate fragments to analyze by MS and characterize linkage types.^281^

In addition to MS methods, antibodies and TUBEs exist which are ubiquitin linkage-specific, and these are used in some studies to validate whether the targeted protein has been ubiquitinated with K48 or K11 chains for degradation.^282^ Simple SDS-PAGE analysis can also be applied; for example, the loss of ubiquitin chains (visualized by a polyubiquitin ladder or smear upon gel staining) on addition of a K48-specific deubiquitinases such as OTUB1 can demonstrate the presence of K48 chains. In fact, commercial toolkits such as UbiCRest combining panels of linkage-specific DUBs can be used with gel-based analysis to help profile ubiquitin linkage preferences *in vitro*.^283^

While numerous tools have been developed, determining the exact molecular profile of ubiquitination, including the architecture of ubiquitin chains, remains challenging. Newly discovered noncanonical ‘beyond lysine’ ubiquitination events and mechanisms in the UPS system have expanded the breadth and scope of the ubiquitin code even further,^284^ and novel methods to study these events will contribute to degrader development studies and campaigns.

## 6 Small Molecule-Mediated Degradation

### 6.1 Methods to Monitor Target Protein Degradation Rate and Levels

A degrader’s efficacy is ultimately determined by its ability to degrade the target protein; thus, having robust experimental methods to evaluate target levels is a critical part of the degrader development pipeline.

The most used method to assess protein degradation is degrader cellular treatment followed by Western blotting. In fact, a bibliographic study in 2023 revealed that, among the journals assessed, 60% of TPD-related papers included Western blot as the only way to evaluate target protein degradation.^285^ This method is semiquantitative, and presents several caveats, most notably that signal saturation is time-consuming, requires well-validated and effective antibodies against the target protein, and requires specific time points to be taken. Therefore, more sensitive and quantitative methods that also allow for continuous readouts in real-time are highly desirable to increase throughput and paint a more complete and accurate picture of protein degradation.

Several degradation-monitoring methods relying on immunoblotting are applied in the context of TPD. Capillary electrophoresis has been performed, providing a higher throughput than Western blot, but with similar drawbacks.^89,286^ Enzyme-linked immunosorbent assay (ELISA) has increased throughput in a plate format and is more quantitative; however, it first requires preparation of labeled antibodies.^163,287,288^ AlphaLISA, TR-FRET, and Lumit assays can be set up in a high-throughput plate-based format and require two different antibodies binding to different epitopes on the target protein, and the assay can only be performed in an end-point manner requiring cell lysis.^289,290^ For TR-FRET, prelabeled secondary antibodies can be purchased to avoid the need to label primary antibodies.^151^

Mass spectrometry is a sensitive and label-free, but low-throughput, method for evaluating protein degradation. Global proteomics analysis is used to examine the abundance of the targeted protein pre- and post-treatment with the degrader and is an important approach for validating the selectivity of the degrader or reveal off-target effects.^142^ For example, Zorba et al. showed off-target degradation of TEC by their BTK PROTACs;^176^ meanwhile, Wang et al. tested a nonselective Helios degrader and identify a novel protein target ZNF324.^291^ The stable isotope labeling with amino acids in cell culture (SILAC) involves growing cells in isotope-enriched media, allowing for identification and quantification of isotope-labeled proteins; a study by An et al. used this approach to identify a new CRBN-recruiting molecular glue target ZFP91.^292^ A methodology called ‘multiplexed proteome dynamics profiling’ (mPDP) that combines SILAC with isobaric mass tagging, developed by Savitski et al., allowed the authors to determine the off-target proteins of their synthesized VHL-recruiting BET degrader PROTAC.^293^

While the above methods are important approaches widely used for assessing target protein degradation, the development of fluorescent and luminescent labels along with endogenous protein tagging strategies has constituted a powerful advance in allowing protein degradation and resynthesis monitoring in real-time in live cells.^294^ Green fluorescent protein (GFP) has been applied in several contexts, for instance, Brd4,^5,20^ cyclin K,^13^ IKZF1,^20^ and KRAS^G12C^.^253^ Recently, cellular degradation has also been quantified by transiently transfecting HEK293 cells with the N-terminally tagged single BET bromodomain NanoLuc-Brd4^BD2^ which allowed sufficient resolution to observe statistically significant differences in *D*_max_ and DC_50_ among the lysine-to-arginine ubiquitin mutants tested.^69^ Other tags such as enhanced ProLabel (ePL) have also been explored.^295^

At present, the most widely used method for monitoring protein degradation in live cells in degrader-development campaigns is the HiBiT system, which was initially established by Riching et al.^58,74^ This approach involves using CRISPR to tag the endogenous substrate protein with a 11-amino acid HiBiT peptide.^294^ Upon cellular transfection with LgBit (an 18 kDa polypeptide), HiBiT and LgBiT associate to form a NanoBiT complex. Cells are treated with the bench-stable compound endurazine, which undergoes reduction by cellular esterases to form a furimazine species which, in turn, binds the NanoBiT complex. When the active NanoBit enzyme oxidizes furimazine, furimamide is released along with a luminescence signal which can be tracked to monitor the NanoBiT-substrate protein proteasomal degradation (Figure 12 **A**).^58,74^ Initially applied to monitor BET proteins^58^ such as BRD4 (as shown in the illustrated example Figure 12 **B**), the method has undergone widespread application, expanding to numerous systems such as endogenous BET bromodomains,^3,10,58^ ^,^ ^66,141,150^ WDR5,^62^ SMARCA2/4,^296,297^ KRAS,^73,298^ CBP/p300,^299^ TEAD,^300^ and CDK2,^301^ among many others.

**Figure 12:**
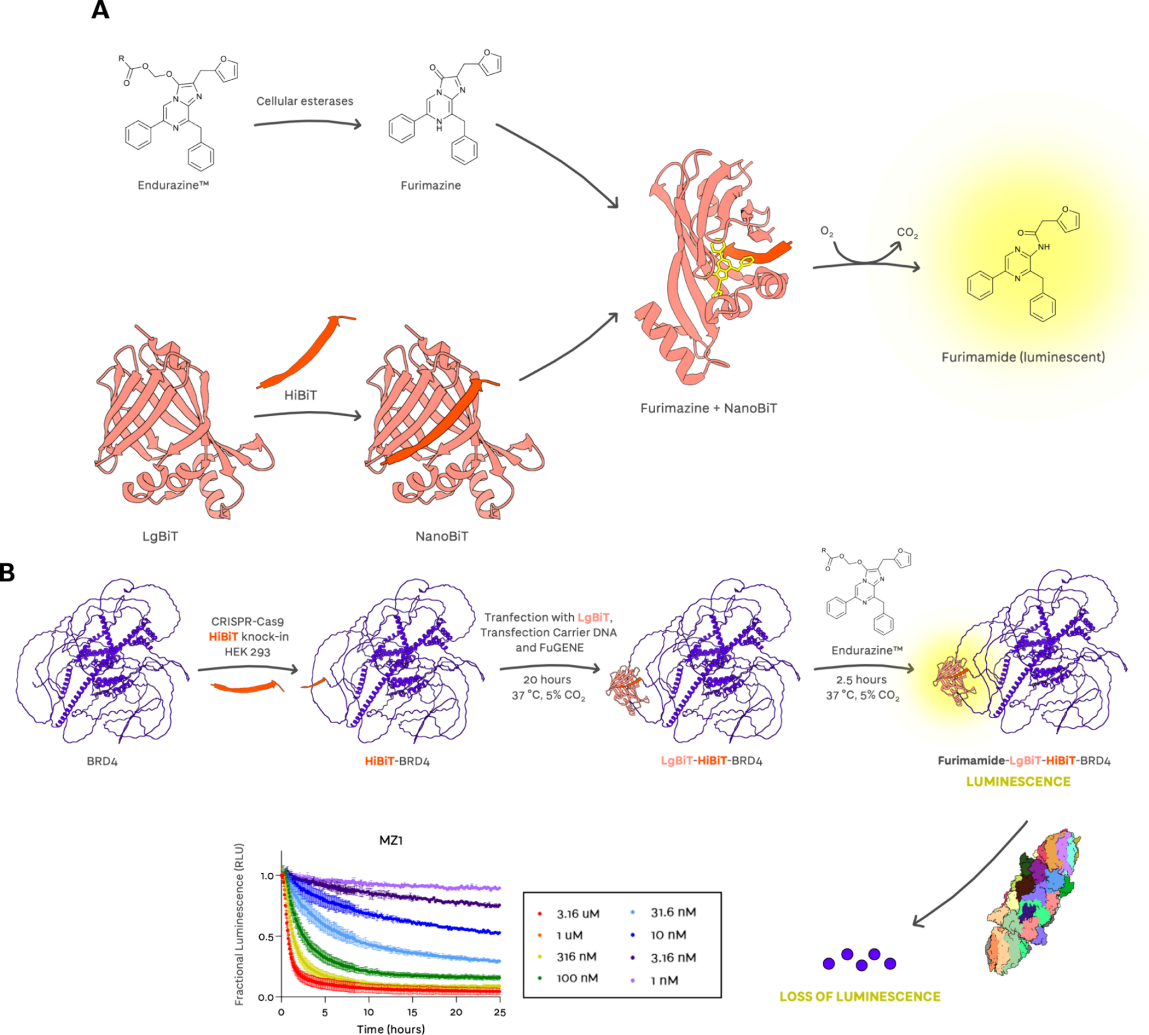
Assay principles for cellular HiBiT degradation with PROTACs. **A** Schematic illustrating how the association of endurazine with LgBit+HiBiT produces the luminescent species detectable in live cell assays. **B** Schematic illustrating an assay workflow for monitoring the degradation of endogenously HiBiT-tagged BRD4.

Currently these protein-tagging strategies provide the most quantitative way of determining the key kinetic parameters of maximal degradation *D*_max_ and half-maximal degradation concentration DC_50_. These methods usually require ectopic or genomic editing of a target cell line, which can prove challenging for certain target proteins when attempted at an endogenous level, e.g., using the CRISPR-Cas system. Moreover, care must be taken to ensure that the tagging does not affect the stability and activity of the native protein.^302^ Additionally, the presence of these tags bearing surface-exposed lysines could be targeted for degradation.^303^ In light of all these caveats and limitations, it remains important to use orthogonal label-free methods of detecting protein levels to validate reporter assay results.

In vitro methods to measure degradation could be explored, for example, through the use of purified 26S proteasome^304^ in the presence of ubiquitination machinery, but thus far this has not been applied to small molecule degrader systems. While some computational machine learning models have been developed to predict target protein degradability, these appear to rely on upstream events such as ubiquitination.^275^ To apply deep learning methods, further experimental data to generate enlarged data sets will be required to train reliable models.^305^

### 6.2 Methods to Study the Target Protein Degradation Pathway and Cellular Localization

In addition to monitoring cellular protein levels, there is a need to understand the pathway through which degradation occurs. For example, crosstalk may exist between different degradation mechanisms such as the UPS system and autophagy.^306,307^ This was the case in a study by Wang et al. where the authors determined that sEH was not degraded in a proteasomal-dependent manner as initially expected but, instead, was subjected to lysosomal degradation.^288^ By using control compounds such as the proteasome inhibitor MG132 or by testing the target protein’s ability to be recognized by p97,^308^ it can be possible to validate parts of the mechanism. Other appropriate controls should be included when testing for target protein degradation, such as the use of inactive compounds which lack binding either to the target protein or to the E3 ligase, to test for degrader-independent protein destabilization or the use of pharmacological blockers of lysosomal/autophagy pathways, such as bafilomycin.^309^

Additional information which is useful for probing the degrader-dependent degradation pathway is knowing the cellular locations of the target protein. Indeed, the UPS system can effectively degrade nuclear and cytosolic proteins,^310^ with some nuclear proteins requiring export to the cytoplasm^311^ to be efficiently degraded by the proteasome.^312^ Certain proteins with other cellular localizations, such as the endoplasmic reticulum, were shown to be transported to the cytoplasm to be destroyed by the UPS.^313^ However, targeting proteins outside of these cellular milieux is not feasible, such as secreted and extracellular proteins, and those which are located in certain organelles such as the mitochondrion. Membrane proteins also constitute attractive targets for TPD. These can either be single-pass, when the polypeptide crosses the lipid bilayer only once, or multipass as is the case when the polypeptide chain can cross multiple times. These proteins typically have a cytoplasmic domain, which would allow access to the UPS machinery, and both classes of membrane protein have been shown to be targeted for degradation by PROTACs.^314–316^ Despite this, the machinery at play in the membrane protein degradation process is still unclear: recent work has suggested that PROTAC treatment and ubiquitination by CRL2^VHL^ could trigger endocytosis and trafficking of EGFR/HER2 to lysosomes for degradation.^317^

## 7 Implications and Applications to Drug Development

Small molecule degraders can be optimized to fully and potently degrade their targeted substrate protein; however, careful evaluation and profiling must be performed to produce a candidate suitable for clinical trials.

Small molecule physicochemical properties directly impact their pharmacokinetic and pharmacodynamic profiles and, therefore, are important for degrader optimization and directly impact their therapeutic profile. Given that degrader molecules follow a catalytic mode of action, a major advantage is that degraders can function at sub-stoichiometric concentrations. Despite this, degrader molecular structures must be fine-tuned to overcome the challenge of absorption and delivery of a sufficient concentration of degrader molecule at the site of action. Degraders can exhibit liabilities in terms of cellular efflux^113,114^ and can be metabolized *in vivo*,^79,318^ leading to excretion or inactivation of the drug molecule. Approaches to improve absorption, metabolism, and efflux must be reconciled with distribution studies, to optimize for tissue specificity and thus reduce cytotoxicity and side-effects in other tissues. The methodology described in Section 2, coupled with *in vivo* studies and proteomics approaches described elsewhere,^34^ can contribute to the production of clinical candidates with calibrated ADME properties.

Even with optimal delivery and target protein engagement, the degrader molecule must still elicit the correct pharmacodynamic response at its site of action – that is, to hijack the UPS mechanism and thereby potently degrade and maintain low levels of the targeted protein. Therefore, optimization and evaluation using the methodology described earlier in the review are equally critical for drug development. Thus, the right balance must be struck to reconcile ADME properties with the desired target degradation response.

Beyond physicochemical properties, targeted delivery strategies and further functionalization of degrader molecules could help to overcome challenges stemming from poor selectivity or permeability. Some approaches which have been proposed are introducing components such as additional target protein ligands,^150,319^ antibodies,^320–322^ tumor cell-specific tags,^323–327^ prodrug moieties,^328^ and cages,^329–331^ modulating delivery with in-cell assembly via click-chemistry^332^ or other vectors,^333^ or controlling degrader activation spatiotemporally with light.^334,335^ Proof-of-concept studies have been published for these polyfunctionalized classes of degraders, but they have not yet produced clinical candidates. Overall, the methodologies discussed in this review (Figure 13), coupled with proteomics analyses and *in vivo* and preclinical evaluation studies, can produce a species suitable for advancement to lead compounds or to the clinic.^73,336^

**Figure 13:**
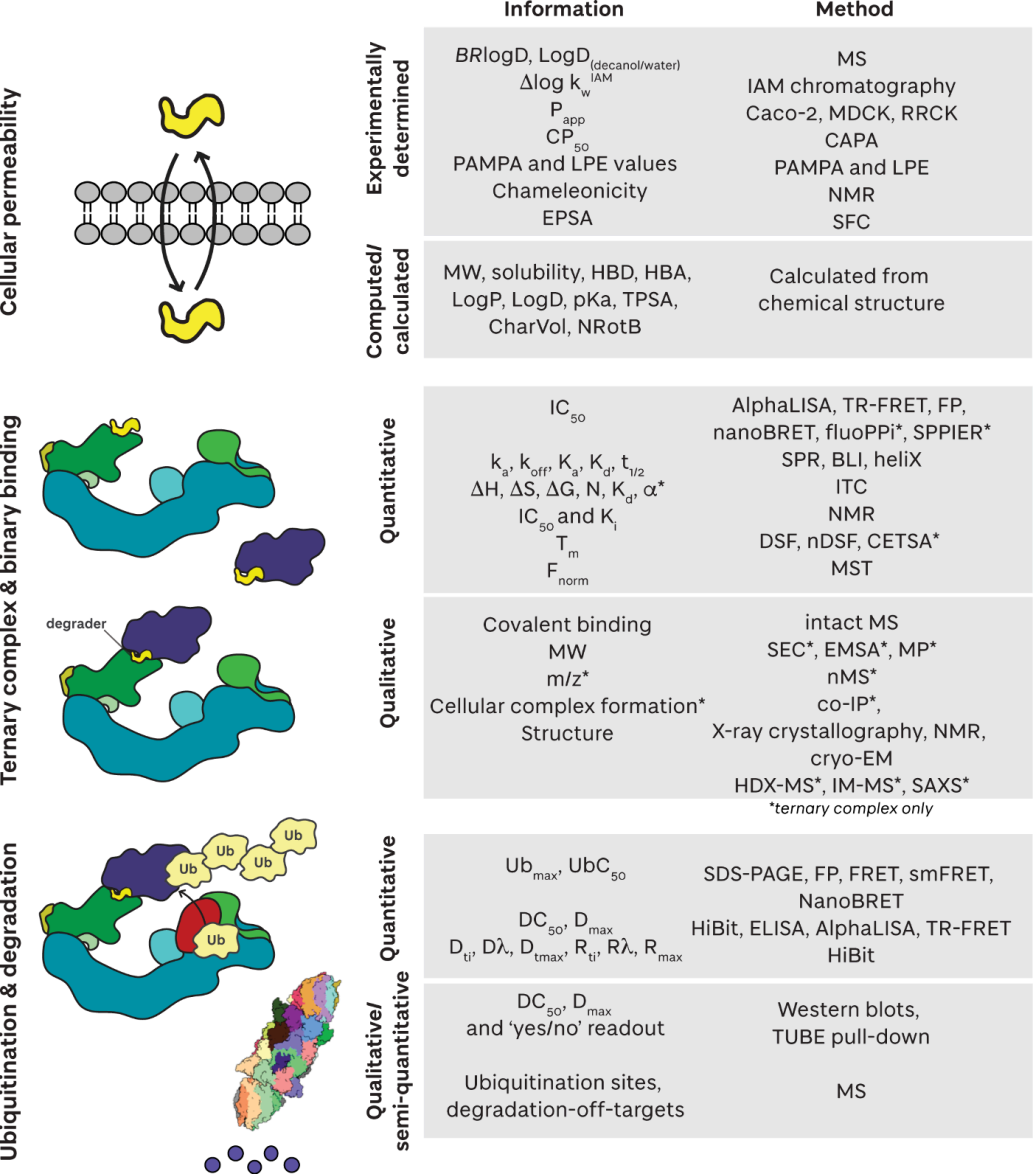
Summary of the parameters and associated methods to assess cellular permeability, binary binding, ternary complex formation, ubiquitination, and degradation.

While this review outlines a comprehensive array of methods, their application in a degrader-development or drug discovery program will be governed by specific considerations when deciding which assays to put in place to meet specific project goals – whether this be target validation, demonstration of cellular degradation, or in vivo proof-of-concept. In practice, this process will involve choosing the right assay for the right purpose and end-goal. For example, to deploy screening cascades, it will be important to consider which assays are best placed in terms of throughput and scalability. In contrast, for in-depth characterization of key compounds or to build detailed mechanistic insights, it will be more important to select assays that provide high-information content and which may illuminate nuances of structure–activity relationships.

In a conventional setting, initial hit identification and SAR to explore chemical space can stem from plate-based high-throughput screening methods, such as HiBiT cellular degradation assays or binary binding biophysical assays. Cellular assays are beneficial, as these capture the target protein in its native environment, and do not require production of large quantities of purified recombinant protein. In recent years, several direct-to-biology protocols have emerged,^204,337,338^ eliminating the need for batch-by-batch molecule purification prior to biological screening. Once binders and initial degrader hits are identified, mechanistic validation is required to confirm the degradation activity goes via the expected UPS-dependent degradation pathway, and medium-throughput assays such as TR-FRET or AlphaLISA can confirm productive ternary complex formation. Finally, a lead optimization phase typically involves a combination of methods from the toolbox to optimize the specific degrader molecule series. Low-throughput but information-rich methods such as X-ray crystallography or cryo-electron microscopy can be particularly powerful to provide the structural information necessary for single-atom or group-based medicinal chemistry modifications to drive potency and rational linker design. Overall, a variety of methods are often combined to solve compound design and optimization challenges.

An illustrative example of PROTAC drug design campaign is the work of Popow et al. developing ACBI3, a pan-KRAS degrader.^73^ The authors started from cocrystal structures of a ligand in the KRAS switch II pocket to identify exit vectors and attached alkyl and PEG linkers to generate a series of VHL-recruiting PROTACs. Using binary and ternary FP screening, the authors identified Compound 2 as highly cooperative and high affinity. Cellular target engagement assays enabled the authors to identify a permeability barrier for Compound 2 and access Compound 3 as a more permeable derivative. Ternary crystal structures of Compound 3 in complex with KRAS^G12 V^ and VCB highlighted opportunities for linker optimization, leading to Compound 4. *In vivo* profiling finally allowed access to ACBI3. More recently, Vetma et al. extended this study through an iterative series of key ternary complex SPR characterization and HiBiT cellular degradation assays, resulting in the development of ACBI4, a pan-KRAS­(on) degrader (Figure 14 **A**).^298^

**Figure 14:**
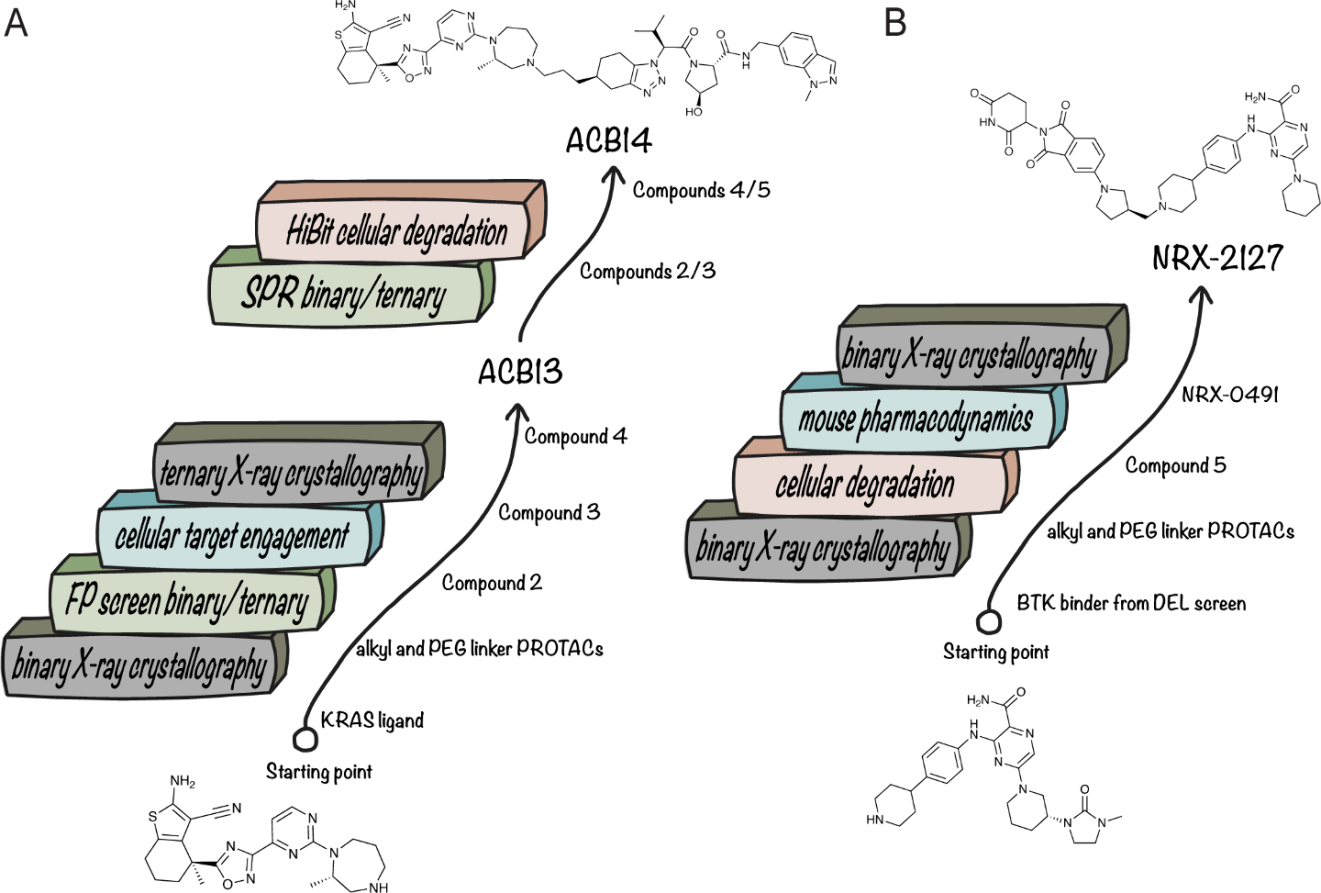
Illustration of the key methods which informed on medicinal chemistry decisions to access intermediate and lead compounds.

An additional example of how a combination of methods can inform medicinal chemistry from an early stage binder hit to lead drug is illustrated by Robbins et al. with the development of clinical candidate NRX-2127 BTK degrader.^336^ The authors started with a high-throughput DNA encoded library (DEL) screening approach to explore chemical space and identify binary binders for BTK and used a combination of binary X-ray crystallography and molecular docking to determine possible exit vectors. From here, a series of PEG-linker PROTACs were developed and screened in cellular degradation assays. Compound 5 was selected owing to its BTK degradation potency and lower molecular weight. Following mouse pharmacodynamic assays and further cellular profiling, NRX-0492 was prioritized for its favorable *in vitro* and *in vivo* results. From here, binary X-ray crystallography enabled the authors to modify the BTK binder to access NRX-2127 (Figure 14 **B**).^336^

## 8 Conclusion and Future Perspectives

This review provides a comprehensive overview of the current toolbox of methods and assays available to characterize and understand the mechanism of action of small molecule degraders operating through the ubiquitin proteasome system degradation pathway.

We first explore how cellular permeability can be measured directly and through fluorescence-based monitoring. We outline techniques for estimating cellular permeability using compound physicochemical properties and also conformational ensemble prediction of degraders using computational and experimental methods, allowing for an appreciation of compound flexibility and folding. Cellular uptake of degraders, particularly of bifunctional molecules which are typically larger in size and have a large exposed polar surface area, is known to be a limiting criterion in degrader development. Particularly for PROTACs and other degraders which fit the ‘beyond rule-of-five’ descriptor, *in silico* assessment and consideration for physicochemical properties must be a priority before laboratory synthesis, after which a range of assays can be deployed to characterize the molecule’s complex behavior in a cell.

We also explored methods to characterize degrader binary binding to either the target protein or E3 ligase and ternary complex formation. We focused on biochemical and biophysical methods to characterize ternary complex size and oligomerization state, in-solution proximity-based and competition-based methods for screening campaigns, as well as biosensor-based and in-solution direct binding methods. These methods are compared and contrasted in terms of their relative degrees of protein consumption, labeling and immobilization requirements, throughput, ranges of detectable K_d_, and, crucially, the type of information they provide. These *in vitro* biophysical assays have been and continue to prove critical for degrader optimization in terms of maximizing the durability (half-life) and stability and cooperativity of ternary complex formation and dissociation equilibria, providing a wealth of invaluable information that can also inform the prediction of PK/PD relationships. These approaches can be complemented by cellular biophysical binary and ternary complex studies, which, albeit less quantitative, report on engagement with the endogenous full-length proteins. Together these assays provide information on compound cell permeability, export/efflux, sequestration, and target protein compartmentalization.

An arsenal of structural biology techniques can be deployed to study degrader protein complexes. Protein X-ray crystallography provides valuable high-resolution information to guide molecular design of optimized binary and ternary complexes; however, it may give an incomplete view of a heterogeneous system. Additional methods, such as cryo-EM, help elucidate sample heterogeneity and provide useful data on larger complexes, including yielding structures of ternary complexes, while in-solution methods offer orthogonal insights to improve our understanding of these complexes.

Ubiquitination has been less widely studied in the context of TPD but constitutes a critical step in the degrader mode of action. Biophysical and structural methods have more recently been applied to optimize for degrader-mediated target *ubiquitinability.* Degradation methods in cells prove critical to verify the mechanism through which degrader molecules act and determine off-target effects, while some plate-based degradation assays can provide quantitative and higher throughput information for compound profiling and screening.

Overall, this review provides insights into how to study degrader development, from validating their basic mode of action, to optimizing for individual steps in the mechanistic pathway. While each and every one of the different assay strategies described herein could be, in principle, applied to drug discovery campaigns, in reality, limitations in the available time and resources mean that only a small subset of these assays will end up being used at any given time on drug discovery projects, whether in an academic or biopharma industry environment – typically based on consideration of throughput, scalability, speed of data generation, and material consumption. Despite these pragmatic considerations, it is important to also recognize that in-depth characterization of the molecular recognition of E3 ligases and target proteins and its downstream impact on target ubiquitination and degradation has in many cases proven critical for the success of degrader molecules. This enablement requires the development and application of *in vitro* and cellular biochemical, biophysical, and structural methods that are often expensive, time-consuming, technically demanding, and low-throughput. Such methods are therefore often best applied to a small selection of carefully chosen compounds, and the challenge is to obtain data within a time scale of a design-make-test-analyze (DMTA) cycle that still allows to inform and guide the medicinal chemist’s decision-making process. Further innovation in methodology, including automation, screening, and computational approaches, will help unlock faster and perhaps deeper insights into degrader action.

The majority of the literature cited here has focused on CRL2^VHL^ and CRL4^CRBN^, and with the repertoire of E3 ligases currently expanding, an increasing number of degrader molecules are being developed to hijack Cullin RING ligases beyond VHL and CRBN.^1004^ While CRL2^VHL^ and CRL4^CRBN^ systems are well-established, much of the methodology discussed here can be applied to degrader development campaigns with other E3 ligases and to other proteins that are targeted for degradation. It is anticipated that the methods discussed in this review will inspire further degrader characterization in the context of their mechanism of action and overall properties and performance and will guide optimization of improved degrader drugs as well as other induced proximity pharmacologies beyond TPD.

## Abbreviations

α: cooperativity
Å: Angstrom
AlphaLISA: amplified luminescent proximity homogeneous assay-linked immunosorbent assay
BET: Bromodomain and Extra-Terminal motif
BD: bromodomain
BIRC: baculoviral IAP repeat containing protein
BLI: Biolayer interferometry
Brd: bromodomain-containing protein
BRET: bioluminescence resonance energy transfer
BRlogD: biologically relevant logD
BTK: Bruton’s tyrosine kinase
CAPA: chloroalkane penetration assay
CDK: cyclin-dependent kinase
CDO1: cysteine dioxygenase type 1
CETSA: cellular thermal shift assay
Chamelogk: chameleonicity metric
CharVol: characteristic volume
CHI: chromatographic hydrophobicity index
cIAP: cellular inhibitor of apoptosis protein
CRBN: cereblon
CRL2^VHL^: VHL cullin 2 RING E3 ubiquitin ligase
CRL4^CRBN^: CRBN cullin 4 RING E3 ubiquitin ligase
cryo-EM: cryo-electron microscopy
Cul: Cullin protein
ΔH: enthalpy
ΔG: free energy
ΔS: entropy
Da: Dalton(s)
DDA1: DET1 and DDB1 associated protein 1
DDB1: damage specific DNA binding protein 1
DC50: half-maximal degradation constant
DCAF: DDB1 and Cul4 associated factor
DLS: dynamic light scattering
*D*_max_: maximum degradation efficacy value
DNA: DNA
DSF: differential scanning fluorimetry
E1: E1 ubiquitin-activating enzymes
E2: E2 ubiquitin-conjugating enzymes
E3: E3 ubiquitin ligases
eloB: elongin B
eloC: elongin C
EMSA: electrophoretic mobility shift assay
EPSA: experimental polar surface area
ER: estrogen receptor
FEM1C: FEM-1 homologue C protein
FIDA: flow-induced dispersion analysis
FITC: fluorescein
fluoPPi: fluorescent PPi
FP: fluorescence polarization
FPS: fluorescence proximity sensing
FRET: Förster resonance energy transfer
GFP: green fluorescent protein
GSPT1: G1 to S phase transition protein 1
GST: glutathione S-transferase
HADDOCK: high-ambiguity driven docking
HaloTag: haloalkane dehalogenase-tagged
HBA: and hydrogen bond acceptor
HBD: number of hydrogen bond donor
HDX-MS: hydrogen–deuterium exchange mass spectrometry
HEK: human embryonic kidney
HIF-1: hypoxia inducible factor 1
HPLC: high-performance liquid chromatography
IAM: immobilized artificial membrane
IC50: half-maximal concentration of inhibition
ITC: isothermal calorimetry
Ka: association constant
*K*_d_: dissociation constant
*K*_i_: inhibitor constant
KLHDC2: Kelch domain-containing protein 2
koff: off-rate
kon: on-rate
LC/MS: liquid chromatography/mass spectrometry
LED: light-emitting diode
Log D: lipophilicity distribution coefficient of ionizable compounds
LogP: lipophilicity partition coefficient
LPE: lipophilic permeability efficiency
LRRK2: leucine rich repeat kinase 2
MDCK: Madin-Darby canine kidney
MST: microscale thermophoresis
MW: molecular weight
*m*/*z*: mass-to-charge ratio
N: stoichiometry coefficient
NanoLuc: nanoluciferase
nDSF: nanoDSF
NMR: nuclear magnetic resonance
nMS: native mass spectrometry
NRotB: number of rotatable bonds
P: polarity
PAMPA: parallel artificial membrane permeability assay
Papp: apparent permeability
PPi: protein–protein interaction
PROTAC: proteolysis targeting chimera
PSSM: position-specific scoring matrix
PWM: positional weighted matrix
RBM39: RNA-binding motif protein 39
RBR: RING-Between-RING
Rbx: RING box protein
RF: random forest
RING: really interesting new gene
RRCK: MDCK-clone Ralph-Russ canine kidney
S: solubility
SAR: structure–activity relationships
SASA: solvent-accessible surface area
SAXS: small-angle X-ray scattering
SDS-PAGE: sodium dodecyl sulfate-polyacrylamide gel electrophoresis
SEC: size exclusion chromatography
SILAC: stable isotope labeling by amino acids in cell culture
smFRET: single molecule FRET
SOCS: suppressor of cytokine signaling
SPPIER: separation of phases-based protein interaction reporter
SpS: spectral shift assay
SPR: surface plasmon resonance
ssDNA: single-stranded DNA
STAT: signal transducer and activator of transcription
t1/2: half-life
TAMRA: tetramethylrhodamine
TDA: thermal denaturation assay
TEC: tyrosine protein kinase
TPD: targeted protein degradation
TPSA: total polar surface area
TR-FRET: time-resolved Förster resonance energy transfer
TRIP12: thyroid hormone receptor interactor 12
TSA: thermal shift assays
TUBE: tandem ubiquitin binding entity
Ub: ubiquitin
UbC50: half-maximal concentration of ubiquitination
Ubmax: maximum conversion to ubiquitinated substrate
UPS: ubiquitin proteasome system
VCB: pVHL-eloB-eloC complex
VHL: von Hippel-Lindau
WDR5: WD repeat-containing protein 5

## Notes

On May 1, 2026, the U.S. Food and Drug Administration (FDA) approved vepdegestrant (ARV-471), an estrogen receptor degrader, for the treatment of patients with specific types of breast cancer. ARV-471 represents the first-ever FDA-approved PROTAC protein degrader.
